# Going Deeper into High and Low Phylogenetic Relationships of Protura

**DOI:** 10.3390/genes10040292

**Published:** 2019-04-10

**Authors:** Antonio Carapelli, Yun Bu, Wan-Jun Chen, Francesco Nardi, Chiara Leo, Francesco Frati, Yun-Xia Luan

**Affiliations:** 1 Department of Life Sciences, University of Siena, Via A. Moro 2, 53100 Siena, Italy; francesco.nardi@unisi.it (F.N.); leo6@student.unisi.it (C.L.); francesco.frati@unisi.it (F.F.); 2 Natural History Research Center, Shanghai Natural History Museum, Shanghai Science & Technology Museum, Shanghai 200041, China; buy@sstm.org.cn; 3 Key Laboratory of Insect Developmental and Evolutionary Biology, Institute of Plant Physiology and Ecology, Shanghai Institutes for Biological Sciences, Chinese Academy of Sciences, Shanghai 200032, China; puppylxr@126.com; 4 Guangdong Provincial Key Laboratory of Insect Developmental Biology and Applied Technology, Institute of Insect Science and Technology, School of Life Sciences, South China Normal University, Guangzhou 510631, China

**Keywords:** basal hexapods, phylogeny, coneheads, mitogenomics, species-delimitation, Pancrustacea

## Abstract

Proturans are small, wingless, soil-dwelling arthropods, generally associated with the early diversification of Hexapoda. Their bizarre morphology, together with conflicting results of molecular studies, has nevertheless made their classification ambiguous. Furthermore, their limited dispersal capability (due to the primarily absence of wings) and their euedaphic lifestyle have greatly complicated species-level identification. Mitochondrial and nuclear markers have been applied herein to investigate and summarize proturan systematics at different hierarchical levels. Two new mitochondrial genomes are described and included in a phylum-level phylogenetic analysis, but the position of Protura could not be resolved with confidence due to an accelerated rate of substitution and extensive gene rearrangements. Mitochondrial and nuclear loci were also applied in order to revise the intra-class systematics, recovering three proturan orders and most of the families/subfamilies included as monophyletic, with the exception of the subfamily Acerentominae. At the species level, most morphologically described species were confirmed using molecular markers, with some exceptions, and the advantages of including nuclear, as well as mitochondrial, markers and morphology are discussed. At all levels, an enlarged taxon sampling and the integration of data from different sources may be of significant help in solving open questions that still persist on the evolutionary history of Protura.

## 1. Introduction

One of the most fascinating and still largely unknown events in the evolution of arthropods is represented by the early differentiation of hexapods. Regrettably, studies on the past and recent evolutionary history of some early diverging six-legged lineages largely rely on revisions produced by a restricted number of specialists of key groups, whose efforts are partly impeded by the high number of unique (autoapomorphic) characters displayed by these taxa, while an overall consensus is still missing. Furthermore, if compared with more recently differentiated lineages (i.e., true insect groups), the fossil record of early diverging hexapods is underrepresented at best, if not completely missing. Combined morphological and molecular analyses have shed some light on the systematics of most high-ranking hexapod groups, but gaps are yet to be filled for the early diverging lineages (e.g., Collembola, Protura, Diplura, Microcoryphia and Zygentoma) [1]. In this respect, molecular studies for the enigmatic group Protura only began in the last few years. Such efforts have addressed the taxonomy of the group at the family level with some success, whereas phylogenetic relationships and species composition are far from clarified.

Proturans are tiny soil-dwelling arthropods, with ~800 species recorded worldwide [2,3,4,5]. Due to their very distinctive and enigmatic morphology, i.e., the primitive lack of antennae and wings, the absence of eyes and tentorium (internal skeleton of the head) and the anamorphic post-embryonic development, their phylogenetic position within the hexapod tree is still debated [6,7,8,9]. Traditionally, Protura were considered an order of class Insecta, associated with Collembola in the group Ellipura [8,10,11,12]. However, the monophyly of Ellipura has been criticized on morphological grounds [13,14,15,16,17] and has recently been rejected by some molecular phylogenetic analyses [18,19,20,21].

By comparing morphological, sperm and developmental characters of Protura, insects and other arthropods, Yin proposed a class rank for Protura [9]. This author further suggested they may have differentiated earlier than is traditionally believed, and that the group may share a more recent common ancestor with some (unidentified) terrestrial groups of chelicerates, myriapods or crustaceans than it does with other six-legged invertebrates, a hypothesis that has found additional support from embryonic development data [22]. Recent studies based on the analysis of molecular data have suggested that Protura may be sister group to Diplura, with which they are associated in the taxon Nonoculata [18,19,20,21], although the monophyly of this clade is still debated because of the extremely divergent morphological characters observed in the two groups. Complete mitochondrial genome sequences (mitogenomes or mtDNAs) of Protura have been studied by [23] in an attempt to define the position of the taxon within Pancrustacea, filling the gap left open by previous analyses that were inclusive of all major early diverging hexapod lineages except proturans [24]. In this respect, both gene order and DNA sequence data have been used, leading, nevertheless, to ambiguous results. On one hand, the position of the two genes encoding for the Leucine transfer RNA (tRNA) along the genome of *Sinentomon erythranum* (Sinentomidae; the first proturan mitochondrial genome available) was similar to that considered ancestral for arthropods but different from what is believed to be the ground plan of Pancrustacea [25], therefore suggesting the placement of Protura outside these latter [23]. On the other hand, phylogenetic analyses based on aligned mitochondrial genes produced clearly untenable phylogenetic reconstructions, possibly due to shared nucleotide compositional bias(es) and/or long branch attraction between the proturan sequence and other unrelated taxa.

**Figure 1:**
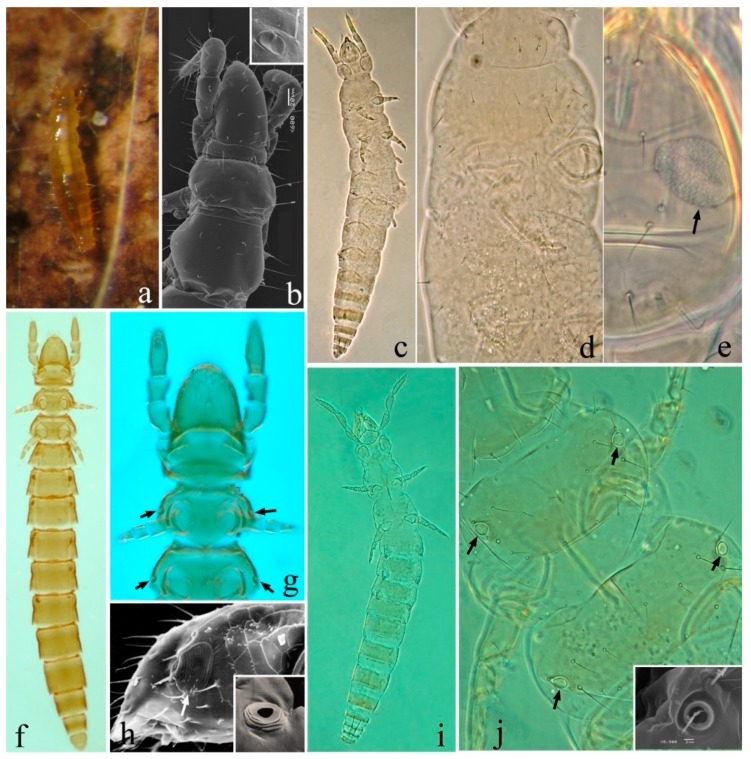
Morphology of Protura. (**a**,**b**) Acerentomata: Acerentomidae (*sensu* [2]). (**a**) *Acerentomon microrhinus*, (**b**) dorsal view of *Baculentulus tianmushanensis* and pseudoculus (inset); (**c**–**e**) *Fujientomon dicestum* (Sinentomata: Fujientomidae), **c.** whole body, (**d**) dorsal view of thorax showing notum, (**e**) right side of head, large pseudoculus (arrow); (**f**–**h**) *Sinentomon erythranum* (Sinentomata: Sinentomidae). (**f**) whole body, (**g**) dorsal view showing notum and spiracles (arrows) (**h**) lateral view of head showing large pseudoculus (arrow) and spiracle (inset); (**i**,**j**) *Eosentomon sakura* (Eosentomata: Eosentomidae). (**i**) ventral view, (**j**) dorsal view of thorax with spiracles (arrows), inset shows detail of spiracle.

Morphological data from alpha taxonomy and ultrastructural observations have been combined to define within-class relationships, with remarkable attempts being undertaken to represent ancestral as well as more recent lineage diversifications [26]. Some of these studies are based on the interpretation of characters that are believed to be connected with the adaptation of Protura to a terrestrial environment, with the presence of tracheae as a key morphological character associated with terrestrialization. In early diverging hexapods, the tracheal system is similar to true insects, with the only exceptions of Protura and Collembola. Tracheae are variously present/absent in proturan families, and it is still unclear whether they represent a plesiomorphic or an apomorphic character for the group. Two alternative classification systems have been proposed for Protura, one by Yin [27,28] and another by Szeptycki [2]. The system of Szeptycki [2] includes some modification with respect to Yin [27,28] concerning the order Acerentomata. Four families of the latter (Acerellidae, Acerentomidae, Berberentulidae and Nipponentomidae) are incorporated into a single family Acerentomidae. In addition, subfamily Acerentulinae *sensu* Yin [27,28] is included by Szeptycki [2] into subfamily Berberentulinae. Considering that the two competing hypotheses, while different in term of taxonomy, have no impact on the phylogenetic tree (but see discussion section), we referred to both systems [2,27,28] as appropriate throughout the text using a slash (on the left side *sensu* [27,28]; on the right side, *sensu* [2]). Protura include three orders (Figure 1): Acerentomata, with three or six families (according to [5] and [27,28], respectively), all without tracheal system (Figure 1a,b); Sinentomata, including family Sinentomidae with tracheal system (Figure 1f–h) and Fujientomidae without tracheae (Figure 1c–e); Eosentomata, including the large family Eosentomidae, with tracheal system (Figure 1i,j), as well as the smaller family Antelientomidae without tracheal system [29]. Additional morphological studies of Protura were based on the analysis of mouthparts, sensilla on foretarsus, chaetotaxy of abdominal appendages, openings of abdominal glands and shape of external genitalia. Results from these studies suggested that the Eosentomata may be considered a primitive taxon, while Acerentomata would be the most recently differentiated group [11]. A competing hypothesis, with Eosentomata regarded as more derived with respect to Acerentomata, nevertheless found support in evidence from post-embryonic development and sperm structure [9,28]. Altogether, the phylogenetic relationships between these three major groups still appear to be an unsolved problem for systematists.

Significant uncertainties remain over phylogenetic and taxonomic relationships at intermediate hierarchical levels as well. The position of Sinentomidae is debated, as this family shares some characters with both Acerentomata and Eosentomata, and, compared to other families, displays an extremely thick cuticle that is difficult to interpret [9,30,31]. Genus *Fujientomon* was initially placed in the Protentomidae by [32], but later proposed for a family rank by [9] and joined to Sinentomidae within Sinentomata. In a modern taxonomic system, these two families are generally associated based on a similar sperm ultrastructure and similar shape of pseudoculi [33] (Figure 1c–g). In addition, the monophyletic status of family/subfamilies Acerellidae/Acerellinae, Acerentomidae/Acerentominae, Berbentulidae/Berberentulinae and Nipponentomidae/Nipponentominae [29] is disputed.

At the species level, barcode approaches have been applied to delimit species boundaries among proturan species using both mitochondrial and nuclear markers [34,35,36], and non-destructive DNA extraction methods have been applied to compare morphological with molecular data [5,37]. Markers of choice were the barcode fragment of *cox1* (cytochrome *c* oxidase subunit 1) and parts of the nuclear ribosomal small (*18S*) and large (*28S*) subunits. Some of these studies included a limited sampling and were essentially focused on the barcoding of selected species, whereas some have also attempted to provide a phylogenetic reconstruction for some families [34,35].

In this study, we analyze the molecular features of two new mitogenomes obtained from the proturan species *Acerella muscorum* (Acerellidae/Acerellinae) and *Acerentomon microrhinus* (Acerentomidae/Acerentominae) and use these data to test whether nucleotide sequences and gene order arrangements are informative with respect to the placement of Protura within the arthropod tree. We further use an extended data set, including the nearly complete *18S* and *28S* rDNA and partial mitochondrial *cox1* sequences from representatives of 6/7 families (Acerentomidae, Eosentomidae, Fujientomidae, Hesperentomidae, Protentomidae and Sinentomidae; *sensu* [2]) to expand over previous studies [18,38] and [35] on family level relationships. In addition, we include all the data available in GenBank (https://www.ncbi.nlm.nih.gov/genbank/) and in the Barcode of Life data base (BOLD, http://www.boldsystems.org) and present distance- and tree-based, single- and multi-locus species delimitation analyses to evaluate the efficiency of molecular markers in defining boundaries among species. Results are analyzed in an evolutionary framework and compared with some of the current hypotheses based on morphological data.

## 2. Materials and Methods

### 2.1. Genome Amplification, Sequencing and Annotation

Several specimens of both *A. muscorum* and *A. microrhinus* were sampled in decaying wood near the Castello di Belcaro (Siena, Italy: 43°18′25.31″ N; 11°17′26.56″ E). Following morphological identification, total genomic DNA was extracted from individual specimens of both species using the Wizard® SV Genomic DNA Purification System kit (Promega, Madison, WI, USA). In each species, three short DNA fragments (~400–600 bp) of *cox1*, *cox3* and *cob* were initially amplified and sequenced using universal primer pairs [39,40]. The entire mitochondrial genome was subsequently amplified in three long segments (*cox1*/*cox3*, *cox3*/*cob*, *cob*/*cox1*) using species-specific oligos designed on the aforementioned DNA fragments and sequenced using a primer walking approach (primers available upon request). Short PCR reactions were performed in a GeneAmp^®^ PCR System 2700 (Applied Biosystem, Foster City, CA, USA) in a total volume of 25 μL with: 2.5 μL of DNA extraction (12 ng/μL), 1.25 μL of each primer (10 mM), 2.5 μL of MgCl_2_ (2.5 mM), 2.5 μL of dNTP mix (10 mM), 5 μL of Green GoTaq Flexi Buffer (Promega), 0.125 μL of GoTaq Flexi DNA polymerase (Promega; 5 u/μL) and 9.875 μL of ddH_2_O. The following cycling parameters were applied: an initial denaturation at 95 °C for 5 min, 35 cycles of denaturation at 95 °C for 1 min, annealing at 50 °C for 1 min and extension at 72 °C for 95 s, followed by a final extension at 72 °C for 7 min. Long-PCRs were performed in 25 μL reaction volume, which included 2.5 μl of the DNA sample (15 ng/μL), 1.25 μL of each primer (10 mM), 12.5 μL of GoTaq Long PCR Master Mix (Promega) and 7.5 μL of ddH_2_O. Cycling conditions were: 35 cycles of denaturation at 94 °C for 1 min, annealing at 50 °C for 1 min and elongation at 60 °C for 1 min/kb. PCR products were purified using the Wizard^®^ SV Gel and PCR Clean-Up System kit (Promega, Madison, WI, USA), and recovered DNA concentration was assessed using a Nanodrop ND1000UV vis (NanoDrop Technologies). Amplified fragments were sequenced on both strands using Sanger sequencing on a DNA Analyzer ABI 3730 at the core facility of BioFab Research Laboratory (Rome, Italy). Electropherograms were assembled using Sequencher 4.4.2 (Gene Codes Corporation, Ann Arbor, MI, USA) to produce the almost complete mtDNA of *A. muscorum* (Table 1) and the complete mtDNA of *A. microrhinus* (Table 2). Assembled sequences were submitted to the tRNAs secondary structure prediction online tool ARWEN [41] for tRNA identification. The presence and secondary structures of a subset of tRNA not identified by ARWEN were manually inferred based on genome sequences. Mapping of the 13 mitochondrial protein-coding genes was achieved by comparison with the *S. erythranum* mtDNA annotation and identification of start and stop codons at gene boundaries.

### 2.2. Nucleotide Composition

The software PAUP* [42] was used to compute base frequencies of each mitogenome for the entire J-strand, for collated genes oriented on the same strand and for subsets of these latter (e.g., first, second and third codon positions). Strand asymmetry was calculated with formulas suggested by [43]: AT-skew = [A% − T%]/[A% + T%] and CG-skew = [C% − G%]/[C% + G%].

### 2.3. Mitogenomic Phylogeny

Complete mitochondrial genome sequences of selected taxa were downloaded from GenBank and collated to the two newly sequenced genomes (Supplementary Materials Table S1). Sequences of genes *atp6*, *atp8*, *cox1-3*, *cob* and *nad5* that are in the same orientation in all ingroup taxa were extracted. Genes *nad5* and *cob* of *Epiperipatus biolleyi* (outgroup) were not included, as they are in the opposite orientation. Gene sequences were retro-aligned using Revtrans (v. 1.4; [44]) and retro-alignments were processed with Gblocks (v. 0.91b; [45]) to identify and exclude regions of unstable alignment. Single gene alignments were concatenated to produce a global dataset of 43 sequences by 6099 nucleotide positions that was used for phylogenetic analyses. Translated protein sequences (43 by 2033 residues) were analyzed in MrBayes (v. 3.2; [46]) using protein model MtPan [24] and averaging over all model (mixed option), both with Gamma correction for rate variation. Nucleotide sequences (43 by 4066 nucleotides, following exclusion of third codon positions) were analyzed using MrModeltest (v. 2.3; [47]) under AIC to identify the optimal model of substitution and in MrBayes using the identified GTR + I + Γ model. In all cases, two runs of 4 chains each were continued for 50 million generations, and convergence was assessed using Tracer (v. 1.6; [48]).

### 2.4. Phylogenetic Multi-Locus Analyses

Multi-locus analyses were performed on concatenated *cox1*, *28S* and *18S* sequences. All 427 available proturan *cox1*, *28S* and *18S* sequences were retrieved from GenBank and BOLD. The final data set included 18 proturan species for which information was available for each of the three markers (Table S2). Two outgroup species were included: the dipluran *Lepidocampa weberi* and *Occasjapyx japonicus* (Accession numbers in Table S2).

Each of the three initial gene sets (i.e., *cox1*, *28S* and *18S*) was aligned through the online tool Clustal Omega (https://www.ebi.ac.uk/Tools/msa/clustalo/), trimmed and manually corrected. The three resulting alignments were concatenated using Mesquite 3.51 [49]. The concatenated alignment was processed with Gblocks (w. 0.91b; [45]) to identify and exclude regions of unstable alignment (725 positions).

The resulting data set was analyzed under Bayesian inference (BI) and Maximum Likelihood (ML), as implemented in MrBayes 3.2.2 [46] and in IQ-TREE 1.6 [50]. The data set, encompassing 5667 aligned positions, was divided into five partitions as follows: first, second and third codon positions for *cox1*, the entire *18S* and the entire *28S*. PartitionFinder 2 [51] and ModelFinder [52] were used, for BI and ML, respectively, to identify the most appropriate evolutionary models (BI: GTR + I + Γ for *18S*, *28S* and second codon positions of *cox1*; SYM + I + Γ and HKY + I + Γ for first and third *cox1* codon positions, respectively; ML: TIM3 + F + R3 for *18S* and *28S*; GTR + F + Γ4, GTR + F + I + Γ4 and TPM2u + F + Γ4, for *cox1* first, second and third codon positions, respectively). BI analysis was run with four chains for 10^6^ generations, sampling every 1000 iterations, with the initial 25% of tree topologies discarded as burn-in. ML tree search was performed with default settings of perturbation strength (-pers command) and stop condition (-nstop command); support values were obtained with 1000 replicates of ultrafast bootstrap approximation [53]. The ML analysis was repeated (details as above) on a reduced data set consisting of *18S* and *28S* sequences only, but with the inclusion of *Fujientomon dicestum* as a representative of Fujientomidae to produce a preliminary hypothesis for the placement of this taxon, even of the absence of complete sequence information.

### 2.5. Discovery Methods of Species Delimitation

Species delimitation analyses were performed on the *cox1* data set using different discovery methods based on pairwise distances (e.g., Automatic Barcode Gap Discovery, ABGD) and on Bayesian and Maximum Likelihood phylogenies (e.g., Poisson Tree Process, PTP and Generalized Yule Coalescent Model, GMYC). All the *cox1* proturan sequences available on public databases, 224 sequences from 55 species, were retrieved. After removal of identical haplotypes in DNAcollapser (http://users-birc.au.dk/biopv/php/fabox/dnacollapser.php), sequences were aligned through the online tool Clustal Omega (https://www.ebi.ac.uk/Tools/msa/clustalo/) and alignments were manually edited and trimmed in Mesquite 3.51 [49]. The final data set consisted of 97 *cox1* haplotypes by 504 aligned positions from 51 proturan species (Table S3) and the two outgroups *Folsomia candida* (Collembola, KX351334) and *Occasjapyx japonicus* (Diplura, HQ882833). The *cox1* single-locus data set was initially analyzed using the online tool Automatic Barcode Gap Discovery (ABGD, http://wwwabi.snv.jussieu.fr/public/abgd/abgdweb.html), which uses pairwise distances to define hypothetical species clusters [54] and groups sequences according to a threshold value calculated on the maximum intra-specific and the minimum inter-specific divergence level observed from the data set. The analysis was performed using the Kimura two-parameter (K2P) model, defining priors for minimum and maximum intra-specific divergence as 0.001 and 0.1, respectively, and a gap width of 0.5.

The Poisson Tree Process was applied to the same data set to identify hypothetical species clusters by modelling speciation rates from the number of nucleotide substitutions described by a phylogenetic tree [55]. BI and ML tree search strategies were applied as described above to infer a proturan *cox1* phylogeny. The data set was partitioned into three charsets: first, second and third codon positions of the mitochondrial barcode fragment; and PartitionFinder 2 [51] and ModelFinder [52] were applied as previously described. The single-locus BI analysis was continued with four chains for 10^6^ generations, sampling every 1000 iterations, and 25% of the tree topologies were discarded as burn-in. The ML tree search was performed with a perturbation strength (-pers command) of 0.2 and a stop condition (-nstop command) of 500; support values were obtained with 1000 replicates of ultrafast bootstrap approximation [53]. Both BI and ML topologies were then analyzed using the web server bPTP (Bayesian Poisson Tree Process; https://species.h-its.org/ptp/), including and excluding the two outgroup species. Each analysis was performed for 500,000 Markov Chain Monte Carlo (MCMC) generations and with a burn-in of 0.25.

The Generalized Mixed Yule Coalescent model (GMYC) for species delimitation, that identifies species clusters based on the tree branching pattern [56], was applied to an ultrametric tree obtained using BEAST 2.4.8 [57]. A single partition was defined, and the best model of sequence evolution was identified using PartitionFinder 2 [51]. A strict molecular clock was applied, defining the clock.rate based on the average mutation rate per million year identified in [58], with a coalescent model of constant population size as the tree prior. Two independent MCMC runs were continued for 10^6^ generations, sampling every 1000 iterations. Convergence was assessed using Tracer v 1.7 [48] and, after excluding the initial 25% generations, the two runs were combined using LogCombiner 2.4.8 [57]. The resulting ultrametric topology was used for the single-threshold GMYC analysis using the splits package [59] in R 3.3.2 (R Core Team 2014, http://www.R-project.org/).

### 2.6. Validation Method of Species Delimitation

Incongruences between species as morphologically defined and clusters obtained using the aforementioned bioinformatic discovery methods for species delimitation were further investigated through a validation approach. Species represented by a single sequence and species never challenged by the discovery methods were removed from the validation analyses, leaving 65 sequences from 19 proturan species. The Bayesian Phylogenetics & Phylogeographic (BPP) program was used applying the multispecies coalescent model [60] to confirm/reject the hypothesis of 38 clusters resulting from the discovery approach on the 19 species data set (Supplementary Materials Table S3). Species delimitation and species-tree inference were jointly performed (i.e., speciesdelimitation = 1, speciestree = 1; A11; [60]). Algorithm 0 and the default settings for fine-tuning parameters were used (ε = 5), as well as the species model prior 1 (i.e., uniform probability for rooted trees). The following combinations of θ (ancestral population size) and τ (root age) were applied, both as gamma distributions: (1) θ: G(2:1000), τ: G(2:100); (2) θ: G(2: 100), τ: G(2: 500); (3) θ: G(1: 10), τ: G(1: 10). Analyses were run for 100,000 MCMC generations, with a sampling frequency of 50 and a burn-in of 1000 generations; each analysis was run twice in order to confirm the consistency of the results. The possibility that three species for which extensive sampling/sequence data are available, *Acerentulus exiguus*, *Ionescuellum haybachae* and *Ionescuellum carpaticum*, may be single species characterized by particularly high levels of genetic divergence (as in [34]) or species complexes (as in our species delimitation analysis) was further assessed in a second validation analysis performed using combined *cox1* and *28S* sequences. The same settings described above were applied with θ and τ as gamma distributions: (1) θ: G(2: 1000), τ: G(2: 100); (2) θ: G(1: 10), τ: G(1: 10).

## 3. Results

### 3.1. Molecular Features of Proturan mtDNAs

**Figure 2:**
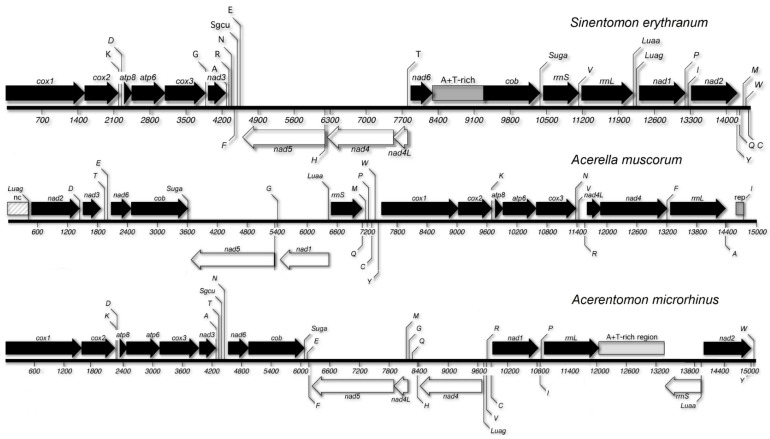
Linearized map of the three proturan mitochondrial genomes. Genes oriented on the J- and N-strand are represented above and below the line, respectively.

The complete, circular, mtDNA sequence of *A. microrhinus* (total length: 15,213 bp) and the almost complete sequence of *A. muscorum* (partial length: 15,018 bp) were determined and deposited in GenBank under accession numbers NC_026666 and NC_026675, respectively. The latter genome is missing part of the A + T-rich region and, probably, *trnSgcu*. In both genomes, as well as in *S. erythranum* [23], most genes are oriented on one strand (25/37 genes for *A. microrhinus*, 27/36 for *A. muscorum* and 29/37 in *S. erythranum*). This strand has been arbitrarily defined as the J-strand (Figure 2).

Canonical start codons (encoding for Methionine) are observed for all *A. microrhinus* protein coding genes (PCGs) and for most of *A. muscorum* PCGs, exceptions being *atp8* and *nad2*, both starting with Isoleucine, *nad5* (Leucine) and *nad3* (Phenylalanine). Incomplete stop codons (T--) are observed in *A. muscorum cob* and *cox2*, as well as *A. microrhinus nad3*, *cob*, *nad5*, *nad4* and *nad4L*. Similarly, both TA- and T-- incomplete stop codons are present in the *S. erythranum* mtDNA [23]. Intergenic spacers, when present, span from 2 to 298 nucleotides for *A. muscorum* (Table 1), and from 1 to 43 nucleotides for *A. microrhinus* (Table 2). Gene overlaps occur in the two genomes both at junctions between genes oriented along the same strand as well as on opposite strands, with a remarkable overlapping region of 62 nucleotides between the J-oriented *trnF* and the N-oriented *trnE* in *A. microrhinus* (Table 2).

The initial 416 nucleotides of the deposited sequence of *A. muscorum* mtDNA, devoid of open reading frames of significant length and including a 61-long tandem repetition of AT dinucleotides, most likely corresponds to a fragment of the A + T-rich region [61]. At variance, no repeats are observed in the *A. microrhinus* A+T-rich region, which is located between the two ribosomial DNA encoding genes (Figure 2).

### 3.2. Nucleotide Composition of mtDNA Strands

Nucleotide composition of proturan mitochondrial genomes is biased towards a higher content of Thymine bases in the J strand (52% in *S. erythranum*, 43% in *A. muscorum* and 39% in *A. microrhinus*) and Adenine nucleotides (39% in *A. muscorum*, 32% in *A. microrhinus* and 25% in *S. erythranum*). Cytosines and Guanines are less frequent, accounting for less than 29% in total. 

**Figure 3:**
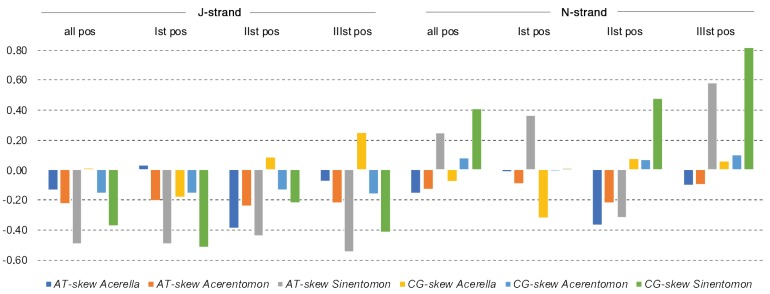
AT- and CG-skew values observed in genes encoded on the J and N strand. Figures are given for concatenated PCGs and separately for each of the three codon positions.

Collectively, these percentages lead to a negative value of AT-skew in *A. microrhinus* (−0.13) and in *A. muscorum*, (−0.08) for the complete J-strand, whereas CG-skew is −0.08 in *A. microrhinus* and 0.02 in *A. muscorum*. For comparison, skew values for the *S. erythranum* J-strand are both highly negative (AT- and CG-skew= −0.35). When different sets of sites are considered (all nucleotides; first, second and third codon positions), AT- and CG-skew values are rarely positive for the J strand (Figure 3), whereas a higher variability is observed for the N-strand. AT-skew values for second codon positions are negative both for J and N strands. Whereas G+T richness of the J-strand is evident and uniformly distributed along the entire genome of *S. erythranum*, this same bias is present but less pronounced in the other two proturan mitochondrial genomes (Supplementary Materials Figure S1). In these latter, the high percentage of Ts calculated for the whole J-strand is mostly dependent on the nucleotide bias (towards a higher percentage of G + T vs A + C content) observed for those genes that are oriented on the same strand, while regions corresponding to genes encoded in the complementary strand show a different trend, i.e., higher to equal percentage of G + T and A + C nucleotides.

### 3.3. TRNA Encoding Genes

Most of the *A. muscorum* tRNA genes display the canonical cloverleaf structure, exceptions being *trnC, trnH, trnLuag* and *trnV* for which one lateral arm is missing (Figure S2). At variance, 11/22 of *A. microrhinus* tRNAs have one incomplete lateral arm (Figure S3). This tendency towards truncated lateral arms has been also reported for *S. erythranum* (18/22 tRNA have one incomplete arm; [23]). Several non-Watson and Crick pairings, the most frequent of which is G•U (26 bonds in *A. muscorum* and 18 in *A. microrhinus*), are present in tRNAs of both species.

### 3.4. Gene Order

Two alternative gene orders, remarkably different from that of both *Limulus polyphemus*, considered as the arthropod ground pattern [62], and *S. erythranum* [23] (Figure 2) have been observed in *A. muscorum* and *A. microrhinus*. Therefore, none of the three sequenced mtDNAs of Protura share a similar gene order. Several unusual rearrangements involve large portions of the Acerentomidae genomes and only three sequence blocks, namely *cox1-cox2-trnK*, *cob-Suga* and *atp8-atp6-cox3*, are shared between all three sequenced proturan mtDNAs and the ancestral gene order for arthropods. In both *Acerella* and *Acerentomon* most of the tRNA-encoding genes appear to be rearranged. Unlike in *Sinentomon*, the two Leucine tRNAs are neither present between *nad1* and *rrnL*, in the position presumed to be ancestral for arthropods, nor in the typical position shared by pancrustacean arthropods (Figure 2). Most of the genes are oriented on one strand, which should therefore be regarded as the J-strand, although strand nucleotide composition data (see above) would suggest that, unlike most arthropods, this is the negative, rather than positive, strand in Protura.

### 3.5. Phylogenetic Analyses of Protura in the Context of Arthropoda

Phylogenetic analyses based on the nucleotide multi-locus mtDNA data set produced conflicting results (Supplementary Materials Figure S4a), while the data set under two different protein models led to superimposable outcomes (Supplementary Materials Figure S4b,c). Support is medium to high at all nodes, with an increase in resolution towards the tips. With the exclusion of Protura, all trees recover a monophyletic Pancrustacea and Ectognatha, with early diverging hexapods being variously clustered with different crustacean groups, and a monophyletic (proteins) or paraphyletic (nucleotides) Myriochelata (Myriapoda + Chelicerata) at their base. The three proturan sequences always form a well-supported monophyletic group, with *Acerentomon* basal to a *Sinentomon* + *Acerella* clade in protein analyses and *Sinentomon* basal to an *Acerentomon* + *Acerella* clade in the nucleotide analysis. In protein analyses, Protura cluster with pycnogonids (3 sequences) in the context of a large clade comprising all Arachnida *plus Symphylella*. In the nucleotide analysis Protura clusters with the dipluran *Campodea* in a near-to-basal position among Pancrustacea, interspersed, alongside Collembola and the dipluran *Japyx*, among different crustacean taxa.

In the two protein analyses both Protura and their sister taxon Pycnogonida are recovered at the end of very long branches (Figure S4b,c), suggesting a possible long branch attraction. Average increase in distance from the basal node compared to the rest of the tree is 1.6×/1.8× for Pycnogonida and 2.6×/2.7× for Protura, based on the mixed and mtPan models, respectively. This does not take place in the nucleotide analysis where Protura are recovered at the end of a long branch, but their sister group is well in the range of other taxa.

### 3.6. Phylogenetic Analyses of Protura

**Figure 4:**
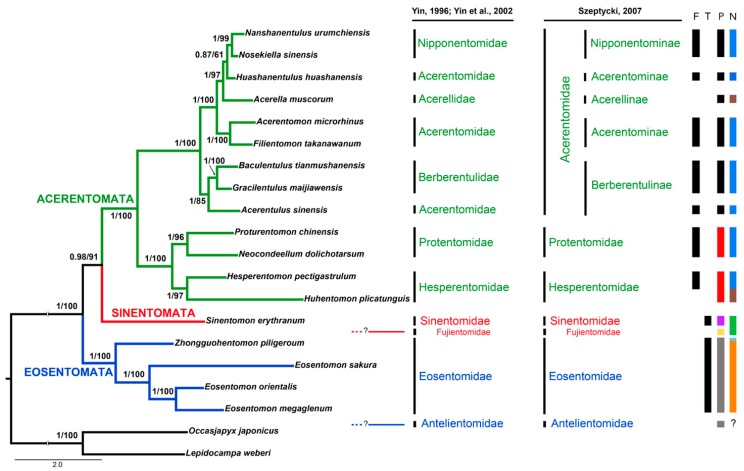
Phylogenetic analysis of Protura based on *cox1, 18S* and *28S* concatenated sequences. Support at nodes is shown as posterior probability/bootstrap. The presence of selected morphological characters is indicated at far right: F, presence of flagellum; T, presence of tracheal system; P, shape of pseudoculi (**black** = peach-shaped; **red** = pear-shaped; **violet** = elliptical; **yellow** = rhomboidal; **gray** = circular); N, shape of nucleus (**blue** = spiral; **brown** = ovoidal; **green** = spherical; light **blue** = dumbbell-like; **orange** = ring-like). The placement of *Fujientomon dicestum* (Fujientomidae) is based on the reduced *18S* and *28S* data set and is to be considered tentative. The most likely position of Antelientomidae (not included in this study, but discussed in the text) is indicated for reference. Both classification systems [2,27,28] used throughout the text are shown.

Inferred phylogenetic trees (both BI and ML), based on multi-locus analyses, both converge to the same topology (Figure 4), with most of the nodes displaying a high statistical support. The two orders Acerentomata and Eosentomata are recovered as monophyletic, as well as all families and subfamilies included in this study. The only exception is represented by the family/subfamily Acerentomidae/Acerentominae ([27,28] and [2], respectively). Early branching of the tree suggests Eosentomata as the most ancient lineage of proturans, sister to a Sinentomata + Acerentomata clade. Within Eosentomata, *Eosentomon sakura* is basal to the cluster *Eosentomon megaglenum* + *Eosentomon orientalis*. Taxon sampling within Sinentomata is limited, as complete data is available for only one species in the family Sinentomidae (*S. erythranum*). Analysis of the reduced dataset (*18S* and *28S* sequences only) recovered a sister group relationship between *S. erythranum* and *F. dicestum*, although with marginal support (Figure 4 and Figure S5), thus suggesting monophyly of Sinentomata. At variance, a richer taxon sampling, that includes all three recognized families, is available for Acerentomata. Their placement highlights a close relationship between Hesperentomidae and Protentomidae, that are recovered in a basal position with respect to the larger cluster Acerentomidae (*sensu* [2]) where Acerentominae is paraphyletic, being split into two lineages, one associated with Nipponentominae and the other diverging earlier among Acerentomata.

### 3.7. Species Delimitation Analysis

The application of molecular discovery methods for species delimitation resulted in a notably higher number of putative proturan species compared to the traditional classification. While the initial single-locus data set included 51 morphologically defined proturan species, the distance-based method ABGD identified 65 putative taxa (Table 3). The questioned species were *A. muscorum*, *Acerentomon dispar*, *Acerentomon italicum*, *A. microrhinus*, *A. exiguus*, *Hesperentomon pectigastrulum*, *I. haybachae* and *Paracerella sinensis* (Table 3). The putative placement for those sequence records lacking a precise species identification (e.g., ‘Eosentomidae sp.’) was considered, but none of these records was identified as belonging to a proturan species included in the present data set. Discovery methods of species delimitation based on a phylogenetic hypothesis (i.e., PTP and GMYC) generally confirmed the result of ABGD, with the exception of few taxa that were further split into additional clusters. In particular, compared to the initial hypothesis of 51 morphologically defined species, PTP identified 69 putative species and GMYC 70. Species that were further questioned by PTP were *A. exiguus*, whose haplotypes were split into three putative species, *P. sinensis*, that was divided into four clusters, and *Hesperentomon bolense* and *I. carpaticum*, that were split into two putative taxa each (Table 3). The only difference between the PTP and GMYC outputs was a new cluster observed within the *A. italicum* complex (Table 3).

Validation analyses performed using BPP were initially limited to species that were questioned by other discovery methods, and for which more than one haplotype was available; hence, 38 putative proturan species were included as the prior hypothesis to be tested (list in Table 3). Generally, the three validation analyses were congruent and further confirmed the results of PTP and GMYC. Specifically, the two analyses with priors θ: G(2: 100), τ: G(2: 500) and θ: G(2: 1,000), τ: G(2: 100) confirmed GMYC results completely, with *A. exiguus* and *A. italicum* split into three lineages and *H. bolense* and *I. carpaticum* divided into two. At variance, the third analysis (θ: G(1: 10), τ: G(1: 10)) produced clusters of 2 and 1 lineages, respectively, but with a lower associated posterior probability (Table 3).

The validation procedure using the combined two-marker data set applied to the three species *A. exiguus*, *I. carpaticum* and *I. haybachae*, already analyzed by [34] and characterized by high levels of genetic diversity suggestive of the presence of putative species complexes, partially but not totally confirmed the results of [34]. In fact, our validation analyses suggested that specimens of *I. carpaticum* sampled from two different Austrian sites may be considered, unlike what was suggested by the single-locus analyses, but in line with [34], a single species (Table 3 and Table 4). On the other hand, the validation analysis suggested the presence of two species among samples referred to as *A. exiguus* and *I. haybachae* (Table 4), differently from what was concluded by [34], which nevertheless noted the high values of genetic divergences (Table 3 and Table 4).

## 4. Discussion

### 4.1. Gene Order and Class Relationships

Mitogenomes of proturan species are somewhat aberrant with respect to other arthropod taxa. One of the most striking features of the group is the high number of gene rearrangements. In this respect, only a limited number of genes clusters are shared among all proturan mitogenomes and it is impossible at present to reconstruct the ground pattern of the group and the rearrangements occurred within and between major lineages. In addition, in all proturans analyzed so far, the *trnLuaa* is not located between *cox1* and *cox2*, as in Pancrustacea, an observation that—if confirmed as not being an autoapomorphy—would suggest the placement of the group outside the pancrustacean clade, questioning the importance of hexapody to support monophyly of Hexapoda in line with authors that, based on alternative character sets, failed to find support for Protura within these latter [13]. However, despite the observation that both *trnLuaa* and *trnLuag* are located in a position similar to that considered ancestral for arthropods (between *nad1* and *rrnL*; [25]) in the *S. erythranum* mtDNA [23], there is no evidence for their placement in the same position in *A. microrhinus* and *A. muscorum*. Therefore, before arguing against a conventional placement of Protura in the context of Arthropoda and prompting for a reinterpretation of the evolution of some morphological characters so far considered autapomorphic for the group (i.e., lack of antennae and post-embryonic development of abdominal tergites), additional gene order data, sufficient to reconstruct their ground pattern with confidence, are necessary. Species of the group Eosentomata, which, according to the phylogenetic reconstruction obtained in this study, appear to diverge early from the group, may have a higher chance of preserving an ancestral genomic asset, and therefore may be more informative in this respect.

### 4.2. Compositional Nucleotide Bias

Nucleotide compositional bias of the J-strand (i.e., a higher content of A vs. T and C vs. G) is a common feature of arthropod lineages, including early diverging hexapod lineages [63,64]. This bias is mostly dependent on asymmetric mutational constraints acting differently on mtDNA strands during the peculiar process of mtDNA replication/transcription [43]. Codon usage of mt-PCGs is also biased due to the necessity of synthetizing hydrophobic polypeptides that will be inserted within the organelle’s inner membrane. In fact, the majority of second codon positions of PCGs, irrespective of the nucleotide bias of the strand where the gene is encoded, are T-rich to guarantee the synthesis of hydrophobic amino acids [43]. Inconsistency between the nucleotide composition of J- and N-strand with respect to other arthropod lineages was, in the past, associated with the reversal of the A + T-rich region [43]. This latter rearrangement may have probably led to the unusual nucleotide content calculated for the *S. erythranum* mtDNA, whereas this effect may be less evident in other lineages.

### 4.3. Aberrant tRNA Genes

Overlaps between tRNA encoding genes are particularly frequent in the Arthropoda mitochondrial genomes. Apart from the well-known lack of a DHU arm in the *trnSgcu* of nearly all Metazoa [65], the occurrence of tRNA genes deprived of lateral arms is a common feature in Chelicerata, Crustacea, Myriapoda and Nematoda [66,67,68], and apparently also in some early diverging hexapod taxa, e.g., Campodeidae (Diplura) [69]. Another feature of adjoining tRNA genes in Protura, shared with many invertebrates, is that they may overlap by some bases, especially when encoded on different strands, while overlaps between tRNAs on the same strand are less frequent, as alternative splicing would be necessary to produce functional molecules. It is unknown whether the high frequency of aberrant tRNAs observed in the three genomes is widespread among Protura; it is also undetermined as to whether some post-transcriptional repair mechanism may restore tRNA structure and functionality (e.g., RNA editing), a phenomenon described in myriapods [62].

### 4.4. Mitogenomic Phylogenetics

The phylogenetic reconstruction, inclusive of three proturan sequences and other arthropodan taxa selected for the common orientation of some mtDNA genes, could not resolve with confidence the relationships of proturan taxa within the arthropod’s tree. According to the amino acid sequence-based analyses (both methods), long branch attraction between Protura sequences and other rapidly evolving lineages with similar nucleotide composition [70] may have produced an artificial Pycnogonida + Protura clade, regardless of their true evolutionary relationships (Figure S4c,d). The nucleotide-based analysis is more in line with other reconstructions, as it clusters Protura with *Campodea fragilis*, but not with the other dipluran species *Japyx solifugus*. Moderate statistical support of nodes connecting Protura to other taxa, as well as the long branches connecting these latter, would definitely suggest caution in the interpretation of the reconstructed phylogeny of the group. In fact, mitochondrial sequence data appear to be unable to establish the relationships of this early diverging hexapod lineage with confidence. Promising alternatives came from large phylogenomic data sets, such as those assembled in the 1Kite project, that nevertheless for the moment include only one single species of Protura, whose placement is not stable across different analyses [21,71]. Mitochondrial gene order studies on Eosentomata may be a further alternative to be contemplated.

### 4.5. Internal Relationships at Inter-Order/Family Levels

According to morphological studies, the monophyly of most families of Protura is not challenged, with some doubts remaining for Acerentomidae. The order Sinentomata is composed of two divergent lineages (Sinentomidae and Fujientomidae), both of which display chaetotaxic patterns that are rather different with respect to other proturan taxa. Their close affinity was suggested based on the similarities observed in the morphology of spermatozoa [27], but with some uncertainties due to other features which are distinctive for each family. With only three species so far described [2] (Figure 1f–g) the taxon Sinentomidae is probably the most peculiar proturan group ever described [30]. Members of the family have thoracic spiracles and tracheal system, large pseudoculi on the head and extremely thick red-brown cuticles. In comparison, Fujientomidae species (only two) do not have spiracles and tracheal system and display extremely large pseudoculi on head and a thin white-yellow cuticle [2] (Figure 1c–e). The close relationship between Sinentomidae and Fujientomidae, though suggestive, does not receive high support in the molecular analysis and *cox1* data are not yet available for the latter. According to [2], Acerentomidae is a large and diverse group of Protura composed of four subfamilies: Acerellinae, Acerentominae, Berberentulinae and Nipponentominae. In the phylogenetic tree (Figure 4), two species of Acerentomidae/Acerentominae (*A. microrhinus* and *Filientomon takanawanum*) cluster together, whereas *Huashanentulus huashanensis* clusters with Nipponentomidae/Nipponentominae. This is not completely at odds with morphology, as species of Acerentomidae/Acerentominae display a large variability and several lineage-specific peculiar characters. Concerning Acerentomidae/Acerentominae the tree topology is inconsistent with both competing classification systems as the group is recovered as polyphyletic. Nevertheless, the inclusion of Acerentulinae *sensu* Yin [27,28] in Berberentulinae [2] is supported by the observation that *Acerentulus sinensis* clusters with *Gracilentulus maijiawensis* and *Baculentulus tianmushanensis*, making Acerentomidae/Acerentominae di- and not triphyletic [2] (Figure 4). A reinterpretation of current taxonomy of the group in the light of the phylogenetic tree shown in Figure 4 highlights Acerentomidae/Acerentominae and Berberentulidae/Berberentulinae as of priority interest for future research.

Unusual sperm cells that lack a motile apparatus and flagellum have been observed in some proturan lineages and this character has been interpreted for taxonomic and phylogenetic purposes [13,14]. Mobile spermatozoa display a peculiar asset of axonemal and acrosomal structures and possess a nucleus whose condensed chromatin appears to be arranged in different shapes. Furthermore, the axoneme of motile spermatozoa lacks central and peripheral microtubules, whereas the number of axonemal doublets is variable (from 9 + 0 to 16 + 0) between families and species. Two evolutionary trends have been observed, with some lineages having long flagellate spermatozoa characterized by a complex acrosome and showing an increased number of axonemal doublets, and some other lineages characterized by short aflagellate sperm deprived of acrosome. Detailed analyses of these reproductive cells led to the conclusion that the motile type (i.e., that observed in Hesperentomidae) should be regarded as the ancestral state, whereas immotile spermatozoon may represent the apomorphic state [14]. Our phylogenetic reconstructions support the opposite view: early diverging orders Eosentomata and Sinentomata include families that have immotile spermatozoa, whereas most Acerentomata (with the notable exception of Acerellidae/Acerellinae) display flagellated spermatozoa (Figure 4). As such, it is suggested that the flagellate motile sperm type may be a derived condition. Curiously, similarities in the external structures of spermatozoa and in microtubular arrangement are observed—if at all—with some Chelicerata groups (e.g., the number of tubulin doublets with Pycnogonidae).

Different shape of pseudoculus and nucleus are additional potentially useful characters to define interfamily relationships among proturans. Pseudoculi may be organized externally in a peach, pear, elliptical, rhomboidal and circular shape. Mapping of these character states along the obtained phylogenetic tree led to the conclusion that a circular shape of pseudoculi, as is observed in Eosentomata, appears to be the plesiomorphic state for the class, whereas elliptical and rhomboidal forms are present in Fujientomidae and Sinentomidae, respectively (Figure 4). The sister groups Hesperentomidae and Protentomidae both have pear-shaped pseudoculi, whereas peach-shaped pseudoculi are exclusive of the remaining Acerentomata. The shape of the nucleus may be also informative. A spiral shaped nucleus is the most frequent model observed in Acerentomata, although within this group, both *Acerella* and *Huhentomon* present an ovoidal nucleus. The ring-like form, as observed in early diverging Eosentomata, is apparently the ancestral state for Protura but not in *Zhongguohentomon*, that in turn displays a dumbbell-like model (Figure 4). Sinentomidae are unique in having a spherical nucleus.

Comparative analyses of tracheal structures of Protura and other early diverging hexapod taxa would suggest independent acquisition of this structures, with the latter displaying the model believed to be more similar to that observed in Pterygota [72]. Despite the unique structure of these respiratory organs in Protura, their occurrence within the taxon is scattered and family specific. According to the most recent evolutionary hypothesis [9,73], tracheae evolved in derived proturan lineages from primitive groups that were deprived of this breathing system. Among the seven or ten families of Protura, only Eosentomidae and Sinentomidae have thoracic spiracles and a tracheal system [29] (Figure 1), whereas the remaining groups exchange gasses using their skin or recta. Therefore, tracheae may have independently evolved twice in Protura: in Eosentomidae and in Sinentomidae. This hypothesis was suggested based on a phylogenetic tree stemming from a reanalysis of the general evolutionary trend of sperm motility observed in Arthropoda—that is, from flagellate to aflagellate [74]—as well as from morphological changes during post-embryo development (e.g., in *S. erythranum* and *Eosentomon* spiracles and tracheae only develop after the first moult) [9,73]. Molecular data, in turn, would suggests the opposite trend (Figure 4), with early diverging lineage Eosentomata (but not Antelientomidae), as well as the Sinentomata family Sinentomidae (but not the related Fujientomidae), displaying a tracheal system that should therefore be considered a plesiomorphic state for the class Protura. If true, tracheae would have apparently been lost at least twice during proturan evolution: once in Acerentomata (for which the lack of tracheae should be considered a synapomorphic state) and a second time in Fujientomidae within Sinentomata (Figure 2). So far, due to the sporadic sampling of specimens from Antelientomidae, no molecular data are available to define the position of this taxon within the proturan tree. Although not completely investigated for their morphological characters, the Antelientomidae are believed to be related with (if not part of) Eosentomata and have no tracheal system and a circular pseudoculus. If the strict relationship of Antelientomidae with Eosentomata will be confirmed, it would also imply that the tracheae may have been lost a third time during proturan diversification. Pattern of evolution of tracheae, if associated with the position of Protura basal to other Hexapoda, would suggest an ancient and possibly shared origin of the tracheal system in all early diverging hexapods, as an adaptation to land environments, followed by the loss of respiratory organs in some lineages of Ellipura due to secondary acquisition of alternative breathing mechanisms.

### 4.6. Overview on Current Proturan Species Delimitation Methods

The analysis of the mitochondrial *cox1* barcode fragment with a distance-based method for proturan species delimitation has already been described in [34]. On a larger dataset, and applying different methods of species delimitation, some of the morphologically determined species included have been confirmed, but some were split into multiple clusters. Conventional taxonomy in poorly studied and morphologically divergent taxa, as well as in taxa characterized by extensive convergent adaptations, is severely impaired by the reduced number of available and clearly interpretable diagnostic characters. Accordingly, the possibility of a correct identification is not devoid of problems, as demonstrated by the inconsistencies between morphological and molecular taxonomies, especially those associated with the occurrence of cryptic species (Table 3). We note that the application of molecular markers and bioinformatic methods for species delimitation may greatly assist the identification of species and lead to a more precise assessment of actual biodiversity. The method that has been most frequently used to confirm the morphological identification of Protura species is based on observed levels of genetic distance among specimens supposed to belong to the same or to different species (e.g., [34,36,75]). However, as observed from Table 3, this approach is not always flawless. Indeed, species like *H. bolense* and *I. carpaticum* have been recovered as the same species when the program ABGD (based on genetic distances) was applied, while all other species delimitation methods have rejected this hypothesis, generally with a high degree of statistical support (Table 3). In other cases (e.g., *A. exiguus*, *A. italicum*, *I. haybachae* or *P. sinensis*), the application of a distance-based method has suggested the occurrence of species complexes (Table 3).

One additional potential drawback associated with the application of tree-based approaches and methods based on the multispecies coalescent is that, in the presence of strong population structure, diverging populations of the same species may be interpreted as separate species, leading to an overestimation of the number of putative species [76]. While this is definitely a matter of concern in Protura, it can be noted that, at this preliminary stage in the application of molecular tools for species delimitation, the groups recovered are nevertheless of primary interest, regardless of their status as largely divergent populations or incipient species, for their possibility to guide further research addressing their distribution, phylogeography and morphology. A formal taxonomic assignment should, in any case, only be based on a combination of multiple evidence, with a central role for morphology. Previous studies have already highlighted substantial genetic divergence among populations of proturan species (e.g., [34,36,75]). Obviously, this aspect can be easily explained by taking into account the euedaphic lifestyle and the absence of wings, features that drastically reduce their dispersal capabilities. Whereas genetic isolation among populations associated with moderate to high levels of genetic divergence is confirmed for species such as *A. exiguus* [34], *H. pectigastrulum* [36] or *P. sinensis* [75], other proturan taxa are an exception. For example, different specimens of *Eosentomon cetium* collected from two Austrian sites 260 km apart displayed genetic distances as little as 1.8% [34], and species delimitation analyses have never questioned their status as the same species (Table 3). Conversely, specimens of *H. bolense* sampled in the same Chinese area showed remarkably high levels of genetic divergence (5% in [36]) and were split into two clusters supported by most of the methods herein applied (Table 3). In this respect, the application of both mitochondrial (*cox1*) and nuclear (*28S*) markers to validation analyses of species delimitation may be the most promising approach, alongside morphological data, to define whether high levels of genetic divergence are a common feature of proturan species or if this taxon is richer in species than so far believed. To test whether the combination of alternative markers may significantly assist with species-level classification of Protura, a validation approach (i.e., BPP) was applied to three key species (i.e., *A. exiguus*, *I. carpaticum* and *I. haybachae*), previously analyzed in [34], that were reported to be characterized by high levels of genetic diversity and identified as complexes of species by single locus delimitation methods (Table 4). Our results confirmed, with a high degree of support, the presence of two putative species within *A. exiguus* and *I. haybachae*, but not within the *I. carpaticum* (Table 4). Considering that in [34] the morphological determination of the *I. haybachae* specimen (PROAT043-12; Table 4) was uncertain, an alternative explanation may be proposed that it actually belongs to a different species of the genus *Ionescuellum*.

Our results, as well as the high levels of genetic distances (21.5%) observed in [34], suggest that more in-depth morphological analyses are required to establish the actual presence of two species within the *A. exiguus* complex. With regards to *I. carpaticum*, both genetic distances (6.2% in [34]) and multi-locus species delimitation point to moderate levels of genetic isolation, likely due to the limited dispersal capabilities of Protura. The same may be applied to *H. bolense*, whose genetic distances were similar to those observed for *I. carpaticum* [34]. Sequencing of the large ribosomal subunit and its application, alongside *cox1*, in a validation multi-locus analysis may further confirm the morphological analysis of [36] and counterbalance the overestimation of the number of species that is sometimes associated with *cox1* single gene analyses. In conclusion, our results suggest, in line with the current prospects of integrative taxonomy [77,78] that the most effective method to separate and identify proturan species with confidence may be a joint combination of different source of biological information (i.e., morphology and multiple molecular markers) together with the use of validation methods (such as, for example, the procedure implemented in the software BPP) that make it possible to test a priori hypotheses that may be based on morphology, geography or other discovery methods of species delimitation [79]. The combination of these methodologies may significantly help in establishing a correct species-level classification of Protura, even in a complex situation characterized by limited background knowledge of their abundance and distribution, limited or difficult to interpret morphological characters and scarce comparative molecular data.

## 5. Conclusions

Our revision of both deep and shallow phylogeny of Protura has highlighted some of the many obstacles that have thus far hampered the possibility of deriving a clear picture of the evolutionary history of the group. Diverging hypotheses are seldom formulated based on different types of data. Furthermore, the limited number of specialists and the peculiar morphological and molecular characteristics associated with this taxon has seriously hindered the development of an overall consensus view. In this respect, the present study, on one hand, summarizes all the information hitherto collected, while on the other highlighting future perspective for the study of Protura evolution and classification. An integrative taxonomy approach, collating data from alternative sources (e.g., molecular, morphological or biogeographical), together with an expanded taxon sampling (e.g., sequencing of complete mitogenomes from Eosentomata, as well as the inclusion in future studies of more molecular and morphological data from species of the three orders), is suggested as the correct strategy for further clarifying hierarchical relationships among Protura at deep, as well as shallow, taxonomic levels.

## Figures and Tables

**Table 1:** Annotation of *Acerella muscorum* mitochondrial genome (partial). Nucleotide composition is calculated on the coding strand; position refers to GenBank accession NC_026675; spacers (positive numbers) and overlaps (negative) are calculated with respect to the following gene.

Genes	A	C	G	T	Length (bp)	Positions	Intergenic Spacers/Overlaps	Strand	Start Codon	Stop Codon
*Luag*	51.61	3.23	6.45	38.71	62	416–477	8	J		
*nad2*	34.91	8.49	7.02	49.58	954	486–1439	4	J	I	TAA
*trnD*	48.39	1.61	6.45	43.55	62	1444–1505	6	J		
*nad3*	32.20	8.19	11.02	48.59	354	1512–1865	70	J	F	TAA
*trnT*	42.19	3.13	10.94	43.75	64	1936–1999	−1	J		
*trnE*	40.32	4.84	8.07	46.77	62	1999–2060	15	J		
*nad6*	36.07	6.97	8.46	48.51	402	2076–2477	4	J	I	TAA
*cob*	32.95	11.37	10.48	45.20	1126	2482–3607	0	J	M	T
*trnSuga*	42.86	4.76	9.52	42.86	63	3608–3670	8	J		
*nad5*	34.90	8.60	9.27	47.23	1662	3679–5340	6	N	L	TAA
*trnH*	37.29	3.39	13.56	45.76	59	5347–5405	−2	N		
*trnG*	45.00	5.00	8.33	41.67	60	5404–5463	−3	J		
*nad1*	33.95	8.54	11.01	46.50	972	5461–6432	−16	N	I	TAA
*trnLuaa*	50.77	0.00	0.00	49.23	65	6417–6481	0	J		
*rrnS*	43.11	7.62	10.05	39.22	617	6482–7098	0	J		
*trnQ*	37.50	3.13	7.81	51.56	64	7099–7162	−3	N		
*trnM*	40.91	13.64	12.12	33.33	66	7160–7225	2	J		
*trnP*	40.32	4.84	8.07	46.77	62	7228–7289	−1	J		
*trnC*	51.85	1.85	3.70	42.59	54	7289–7342	13	N		
*trnW*	47.76	7.46	5.97	38.81	67	7356–7422	−3	J		
*trnY*	36.92	9.23	15.39	38.46	65	7420–7484	5	N		
*cox1*	31.14	13.26	13.52	42.07	1538	7490–9027	0	J	M	TA
*cox2*	37.63	11.09	9.15	42.13	667	9028–9694	5	J	M	T
*trnK*	34.48	15.52	12.07	37.93	58	9700–9757	7	J		
*atp8*	49.36	7.69	1.92	41.03	156	9765–9920	−1	J	I	TAA
*atp6*	35.28	9.87	8.37	46.49	669	9920–10588	−1	J	I	TAA
*cox3*	33.21	11.11	10.99	44.70	792	10588–11379	2	J	M	TAA
*trnN*	47.62	3.18	7.94	41.27	63	11382–11444	−1	J		
*trnV*	48.28	10.35	8.62	32.76	58	11444–11501	60	J		
*trnR*	35.82	13.43	11.94	38.81	67	11562–11628	−25	N		
*nad4L*	36.08	5.16	9.62	49.14	291	11604–11894	−23	J	M	TAA
*nad4*	36.34	9.46	9.69	44.52	1332	11872–13203	2	J	I	TAA
*trnF*	44.62	4.62	10.77	40.00	65	13206–13270	0	J		
*rrnL*	44.38	7.25	7.79	40.58	1104	13271–14374	0	J		
*trnA*	49.23	4.62	9.23	36.92	65	14375–14439	298	N		
*trnI*	34.43	8.20	18.03	39.34	61	14738–14798		J		
*mean*	36.50	9.20	9.76	44.54						

**Table 2:** Annotation of *Acerentomon microrhinus* mitochondrial genome. Nucleotide composition is calculated on the coding strand; position refers to GenBank NC_026666; spacers (positive numbers) and overlaps (negative) are calculated with respect to the following gene.

Genes	A	C	G	T	Length (bp)	Positions	Intergenic Spacers/Overlaps	Strand	Start Codon	Stop Codon
*rrnS*	40.77	11.57	9.09	38.57	726	1–726	0	N		
*trnLuaa*	49.18	4.92	3.28	42.62	61	727–787	−2	N		
*nad2*	28.62	9.64	14.36	47.38	954	786–1739	−3	J	M	TAA
*trnY*	37.10	8.07	16.13	38.71	62	1737–1798	−1	N		
*trnW*	39.71	10.29	8.82	41.18	68	1798–1865	1	J		
*cox1*	25.13	14.45	18.75	41.67	1536	1867–3402	4	J	M	TAA
*cox2*	30.34	12.56	17.19	39.91	669	3407–4075	20	J	M	TAA
*trnK*	46.55	10.35	8.62	34.48	58	4096–4153	−37	J		
*trnD*	41.07	3.57	10.71	44.64	56	4117–4172	7	J		
*atp8*	33.33	9.52	9.52	47.62	147	4180–4326	−13	J	M	TAA
*atp6*	28.00	15.11	17.48	39.41	675	4314–4988	−1	J	M	TAA
*cox3*	27.29	13.71	17.59	41.41	722	4988–5779	12	J	M	TAA
*nad3*	24.77	12.39	16.92	45.92	331	5792–6122	0	J	M	T
*trnA*	46.43	5.36	7.14	41.07	56	6123–6178	0	J		
*trnT*	53.70	1.85	5.56	38.89	54	6179–6232	1	J		
*trnSgcu*	46.55	8.62	6.90	37.93	58	6234–6291	1	J		
*trnN*	49.12	8.77	5.26	36.84	57	6293–6349	28	J		
*nad6*	28.99	6.28	16.43	48.31	414	6378–6791	−1	J	M	TAA
*cob*	26.04	13.20	17.36	43.40	1129	6791–7919	0	J	M	T
*trnSuga*	35.59	6.78	15.25	42.37	59	7920–7978	−1	J		
*trnF*	46.03	6.35	6.35	41.27	63	7978–8040	−62	N		
*trnE*	42.86	6.35	6.35	44.44	63	7979–8041	−1	J		
*nad5*	32.47	15.48	11.91	40.15	1654	8041–9694	6	N	M	T
*nad4L*	33.93	8.57	10.71	46.79	280	9701–9980	5	N	M	T
*trnM*	36.92	15.39	12.31	35.39	65	9986–10050	−2	J		
*trnG*	40.00	16.67	15.00	28.33	60	10049–10108	0	J		
*trnQ*	37.50	3.13	15.63	43.75	64	10109–10172	0	J		
*trnH*	43.86	1.75	12.28	42.11	57	10173–10229	0	N		
*nad4*	29.86	15.77	14.33	40.03	1249	10230–11478	43	N	M	T
*trnLuag*	37.50	8.93	16.07	37.50	56	11522–11577	1	N		
*trnV*	33.90	10.17	5.09	50.85	59	11579–11637	−11	N		
*trnR*	36.00	12.00	12.00	40.00	50	11627–11676	−4	J		
*trnC*	44.83	6.90	6.90	41.38	58	11673–11730	4	N		
*nad1*	25.78	14.02	21.25	38.94	927	11735–12661	1	J	M	TAA
*trnI*	46.77	8.07	11.29	33.87	62	12663–12724	−2	N		
*trnP*	40.00	3.33	13.33	43.33	60	12723–12782	0	J		
*rrnL*	38.52	10.22	12.39	38.88	1106	12783–13888	0	J		
*A+T-rich*	27.40	23.47	16.45	32.68	1325	13889–15213				
*mean*	30.85	13.49	15.01	40.65						

**Table 3:**
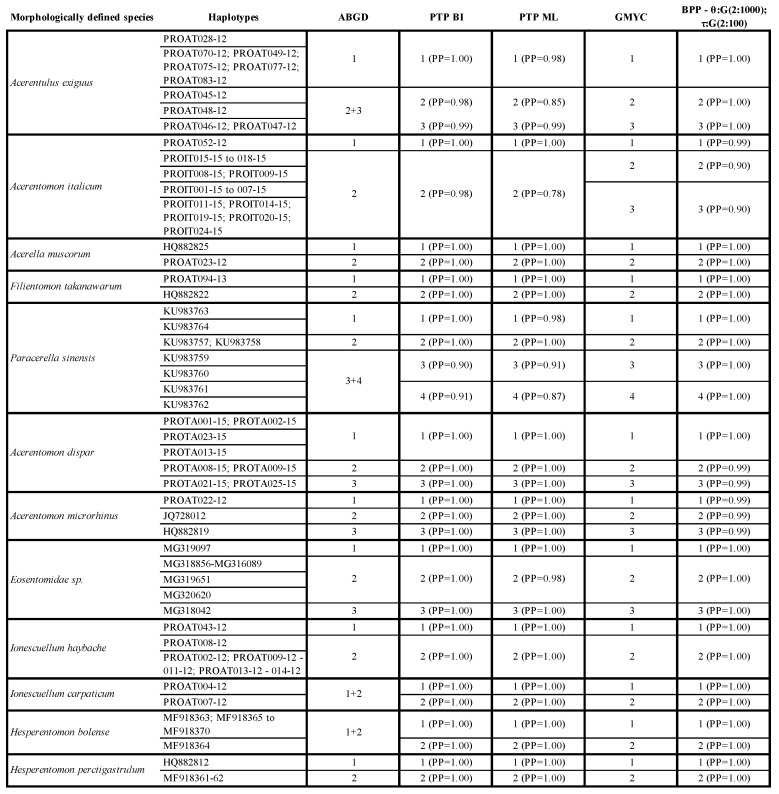
Species delimitation analysis (*cox1*). Only morphological species that have been subdivided in two or more sublineages by at least one species delimitation method are included, see Supplementary Table S3 for full information. First column lists species as morphologically defined following BOLD annotations. Second column reports BOLD identifiers for each specimen; specimens sharing the same *cox1* haplotype (after alignment trimming) are grouped using thin horizontal lines. Columns 3 to 7 show results of different analyses for species delimitation; an open cell indicates a cluster of the corresponding specimens listed in the second column; open cells carry a numerical indication of the corresponding sublineage (for cross table reference) and (where applicable) statistical support for the group expressed as posterior probabilities; in BPP, only results associated with the intermediate parameter combination are shown, see Table S3 for full information.

**Table 4:**
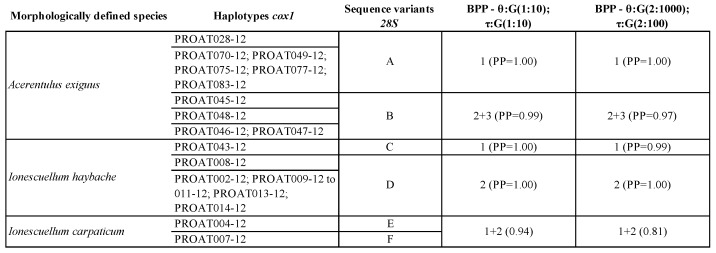
Species delimitation testing (*cox1* and *28S*). First column lists species as morphologically defined following BOLD annotations. Second column reports BOLD identifiers for each specimen; specimens sharing the same *cox1* haplotype (after alignment trimming) are grouped using thin horizontal lines. Third column indicates sharing of *28S* sequence among specimens, see BOLD for sequence data. Columns 3 and 4 show results of BPP testing on species delimitation; an open cell indicates a cluster of the corresponding specimens listed in the second column; open cells carry a numerical indication of the corresponding sublineage (for cross table reference) and statistical support for the group expressed as posterior probabilities.

## Supplementary Materials

The following are available online at https://www.mdpi.com/2073-4425/10/4/292/s1, Figure S1: G+T vs. A+C content, calculated in a sliding window of 100 bp along the J-strand of the genomes of *S. erythranum*, *A. muscorum* and *A. microrhinus*. Genome maps are shown below each graph, Figure S2: Secondary structure of the 21 tRNAs detected in the mitochondrial genome of *A. muscorum*. The *trnSgcu* was not identified, Figure S3: Secondary structure of the 22 tRNAs detected in the mitochondrial genome of *A. microrhinus*, Figure S4: Phylogenetic analysis of Protura in the context of Arthropoda based on a subset of mitochondrial genes found in the same orientation. Posterior probabilities are shown above nodes. a: nucleotide dataset; b: amino acid dataset, model mixed; c: amino acid dataset, model MtPAN, Figure S5: Phylogenetic analysis of Protura based on the reduced *18S* and *28S* data set. Boostrap values shown at nodes, Table S1: List of species included in the phylogenetic analysis of Protura in the context of Arthropoda based on a subset of mitochondrial genes found in the same orientation. GenBank accession numbers are shown, Table S2: List of species included in the phylogenetic analysis of Protura based on *cox1*, *18S* and *28S* concatenated sequences. GenBank accession numbers are shown, Table S3: Species delimitation analysis (*cox1*). First column lists species as morphologically defined following BOLD or GenBank annotations. Second column reports BOLD identifiers for each specimen or GenBank accession for sequences not present in BOLD; specimens sharing the same *cox1* haplotype (after alignment trimming) are grouped using thin horizontal lines. Columns 3 to 9 show results of different analyses for species delimitation; an open cell indicate a cluster of the corresponding specimens listed in the second column; open cells carry a numerical indication of the corresponding sublineage (for cross table reference) and (where applicable) statistical support for the group expressed as posterior probabilities. Open cells with an asterisk indicate alternative clusters with similar support. Grey shading highlights morphological species that have been subdivided in two or more sublineages by at least one species delimitation method.
