# Supplementary material for: Going Deeper into High and Low Phylogenetic Relationships of Protura
Source: Genes (Basel). 2019 Apr 10;10(4):292. doi: 10.3390/genes10040292 (PMC6523364; doi:10.3390/genes10040292)
Supplement: Supplementary file 1 [file genes-10-00292-s001.pdf]

Supplementary Materials: Going Deeper into High and Low Phylogenetic Relationships of Protura

Antonio Carapelli, Yun Bu, Wan-Jun Chen, Francesco Nardi, Chiara Leo, Francesco Frati and Yun-Xia Luan

Table S1. List of species included in the phylogenetic analysis of Protura in the context of Arthropoda based on a subset of mitochondrial genes found in the same orientation. GenBank accession numbers are shown.

**Crustacea**	
Daphnia pulex	NC_000844
Pagurus longicarpus	NC_003058
Triops cancriformis	NC_004465
Pollicipes polymerus	NC_005936
Eriocheir sinensis	NC_006992
Squilla empusa	NC_007444
Tetraclita japonica	NC_008974
**Hexapoda**	
Orthetrum triangulare	AB126005
Grylloblatta sculleni	DQ241796
Timema californicum	DQ241799
Sminthurus viridis	EU016192
Trigoniophthalmus alternatus	EU016193
Cryptopygus antarcticus	EU016194
Atelura formicaria	EU084035
Locusta migratoria	NC_001712
Tricholepidion gertschi	NC_005437
Periplaneta fuliginosa	NC_006076
Nesomachilis australica	NC_006895
Japyx solifugus	NC_007214
Tamolanica tamolana	NC_007702
Campodea fragilis	NC_008233
Acerella muscorum	KJ101608
Acerentomon microrhinus	JQ728012
Sinentomon erythranum	HQ199311
Myriapoda	
Symphylella sp	EF576853
Lithobius forficatus	NC_002629
Narceus annularus	NC_003343
Thyropygus sp	NC_003344
Scutigera coleoptrata	NC_005870
Antrokoreana gracilipes	NC_010221
Chelicerata	
Phalangium opilio	EU523757
Damon diadema	FJ204233
Limulus polyphemus	NC_003057
Ornithodoros moubata	NC_004357
Centruroides limpidus	NC_006896
Achelia bituberculata	NC_009724
Nothopuga sp	NC_009984
Pseudocellus pearsei	NC_009985
Mastigoproctus giganteus	NC_010430
Calisoga longitarsis	NC_010780
Nymphon gracile	NC_008572
Ammothea carolinensis	NC_014671
Outgroup	
Epiperipatus biolleyi	NC_009082

Table S2. List of species included in the phylogenetic analysis of Protura based on *cox1*, *18S* and *28S* concatenated sequences. GenBank accession numbers are shown.

Species	***cox1***		***28S***		***18S***	
	**Accession number**	**Length (bp)**	**Accession number**	**Length (bp)**	**Accession number**	**Length (bp)**
Eosentomon megaglenum	HQ882831	657	EU557268	3670	EU557254	1945
Eosentomon orientale	HQ882830	657	EU557267	3710	EU557253	1946
Acerentulus sinensis	HQ882820	657	EU557260	3739	EU557247	1942
Baculentulus tianmushanensis	HQ882817	657	EF192433	3801	AY037169	1984
Gracilentulus maijiawensis	HQ882818	657	EF192435	3753	EU557246	1935
Filientomon takanawarum	HQ882822	657	EU557261	3766	EU557249	1939
Acerentomon microrhinus	HQ882819	657	HQ882810	3777	HQ882808	1936
Acerella muscorum	HQ882825	657	HQ882811	3774	HQ882809	1942
Huashanentulus huashanensis	HQ882821	657	EU557262	3764	EU557248	1940
Nanshanentulus urumchiensis	HQ882824	657	EU557264	3718	EU557250	1940
Nosekiella sinensis	HQ882823	657	EU557263	3684	EU557251	1942
Zhongguhentomon piligeroum	HQ882828	657	EU557266	3981	EU557252	1943
Proturentomon chinense	HQ882814	657	EU557257	3758	EU557244	1936
Sinentomon erythranum	HQ882826	657	EF192442	4043	AY596358	1934
Huhentomon plicatunguis	HQ882813	657	EU557256	3790	EU557243	1943
Neocondeellum dolichotarsum	HQ882815	657	EU557258	3795	AY037170	1975
Hesperentomon pectigastrulum	HQ882812	657	EU557255	3793	EU557242	1943
Eosentomon sakura	HQ882829	657	EF192434	3789	AY596355	1948
Lepidocampa weberi	NC022675	657	EF192436	3696	AY037167	1878
Occasjapyx japonicus	HQ882833	657	EF192438	4255	AY596365	2040

Table S3. Species delimitation analysis (*cox1*). First column lists species as morphologically defined following BOLD or GenBank annotations. Second column reports BOLD identifiers for each specimen or GenBank accession for sequences not present in BOLD; specimens sharing the same *cox1* haplotype (after alignment trimming) are grouped using thin horizontal lines. Columns 3 to 9 show results of different analyses for species delimitation; an open cell indicate a cluster of the corresponding specimens listed in the second column; open cells carry a numerical indication of the corresponding sublineage (for cross table reference) and (where applicable) statistical support for the group expressed as posterior probabilities. Open cells with an asterisk indicate alternative clusters with similar support. Grey shading highlights morphological species that have been subdivided in two or more sublineages by at least one species delimitation method

Morphologically defined species	Haplotypes	ABGD	PTP BI	PTP ML	GMYC	BPP - θ:G(1:10); τ:G(1:10)	BPP - θ:G(2:100); τ:G(2:500)	BPP - θ:G(2:1000); τ:G(2:100)
Acerentomon doderoi	PROAT026-12; PROAT030-12; PROAT031-12; PROAT034-12; PROAT036-12 to 039-12; PROAT042-12; PROAT054-12; PROAT055-12; PROAT069-12; PROAT071-12	1	1 (PP=0.95)	1 (PP=1.00)	1	1 (PP=1.00)	1 (PP=0.99)	1 (PP=1.00)
	PROAT025-12							
	PROAT029-12; PROAT032-12; PROAT033-12; PROAT035-12; PROAT040-12; PROAT041-12; PROAT063-12 to 066-12							
	PROAT027-12							
Acerentomon maius	PROAT001-12	1	1 (PP=1.00)	1 (PP=0.96)	1	1 (PP=1.00)	1 (PP=0.99)	1 (PP=1.00)
	PROAT016-12							
	PROAT005-12; PROAT019-12; PROAT024-12							
	PROAT020-12							
	PROAT006-12							
Acerentomon carpaticum	PROAT003-15 - PROAT007-15	1	1 (PP=1.00)	1 (PP=1.00)	1	-	-	-
Baculentulus densus	PROAT095-13	1	1 (PP=1.00)	1 (PP=1.00)	1	-	-	-
Acerentulus tuxeni	PROAT051-12; PROAT056-12 to 058-12; PROAT060-12 to 062-12; PROAT072-12; PROAT076-12; PROAT079-12 to 082-12; PROAT084-12; PROAT086-12; PROAT087-12	1	1 (PP=1.00)	1 (PP=0.99)	1	1 (PP=0.98)	1 (PP=0.97)	1 (PP=1.00)
	PROAT059-12							
Acerentulus exiguus	PROAT028-12	1	1 (PP=1.00)	1 (PP=0.98)	1	1 (PP=0.95)	1 (PP=0.96)	1 (PP=1.00)
	PROAT070-12; PROAT049-12; PROAT075-12; PROAT077-12; PROAT083-12							
	PROAT045-12	2+3	2 (PP=0.98)	2 (PP=0.85)	2	* 2+3 (PP=0.43); * 2 (PP=0.52)	2 (PP=0.80)	2 (PP=1.00)
	PROAT048-12							
	PROAT046-12 - 047-12		3 (PP=0.99)	3 (PP=0.99)	3	*3 (PP=0.52)	3 (PP=0.78)	3 (PP=1.00)
Acerentomon italicum	PROAT052-12	1	1 (PP=1.00)	1 (PP=1.00)	1	1 (PP=0.97)	1 (PP=0.95)	1 (PP=0.99)
	PROIT015-15 to 018-15	2	2 (PP=0.98)	2 (PP=0.78)	2	2+3 (PP=0.60)	2 (PP=0.62)	2 (PP=0.90)
	PROIT008-15; PROIT009-15							
	PROIT001-15 to 007-15				3		3 (PP=0.61)	3 (PP=0.90)
	PROIT011-15; PROIT014-15; PROIT019-15; PROIT020-15; PROIT024-15							
Eosentomon sp.	PROAT021-12	1	1 (PP=1.00)	1 (PP=1.00)	1	-	-	-
Eosentomon cetium	PROAT073-12	1	1 (PP=1.00)	1 (PP=0.97)	1	1 (PP=1.00)	1 (PP=0.98)	1 (PP=1.00)
	PROAT068-12							
	PROAT078-12							
	PROAT085-12							
Eosentomon sakura	HQ882829	1	1 (PP=1.00)	1 (PP=1.00)	1	-	-	-
Eosentomon megaglenum	HQ882831	1	1 (PP=1.00)	1 (PP=1.00)	1	-	-	-
Eosentomon orientale	HQ882830	1	1 (PP=1.00)	1 (PP=1.00)	1	-	-	-
Acerella muscorum	HQ882825	1	1 (PP=1.00)	1 (PP=1.00)	1	1 (PP=0.81)	1 (PP=0.94)	1 (PP=1.00)
	PROAT023-12	2	2 (PP=1.00)	2 (PP=1.00)	2	2 (PP=0.81)	2 (PP=0.94)	2 (PP=1.00)
Acerentulus sp. 2	KJ210838-39	1	1 (PP=1.00)	1 (PP=1.00)	1	-	-	-
Filientomon takanawarum	PROAT094-13	1	1 (PP=1.00)	1 (PP=1.00)	1	1 (PP=0.96)	1 (PP=0.95)	1 (PP=1.00)
	HQ882822	2	2 (PP=1.00)	2 (PP=1.00)	2	2 (PP=0.96)	2 (PP=0.95)	2 (PP=1.00)
Acerentulus sp. 1	KJ210841; KJ210842	1	1 (PP=1.00)	1 (PP=1.00)	1	-	-	-
Gracilentulus maijiawensis	HQ882818	1	1 (PP=1.00)	1 (PP=1.00)	1	-	-	-
Yamatentomon guoi	GBA11456-13	1	1 (PP=0.99)	1 (PP=0.99)	1	1 (PP=0.92)	1 (PP=0.96)	1 (PP=1.00)
	GBA11457-13							
Baculentulus tianmushanensis	HQ882817	1	1 (PP=1.00)	1 (PP=1.00)	1	-	-	-
Paracerella sinensis	KU983763	1	1 (PP=1.00)	1 (PP=0.98)	1	1 (PP=0.85)	1 (PP=0.95)	1 (PP=1.00)
	KU983764							
	KU983757; KU983758	2	2 (PP=1.00)	2 (PP=1.00)	2	2 (PP=0.83)	2 (PP=0.93)	2 (PP=1.00)
	KU983759	3+4	3 (PP=0.90)	3 (PP=0.91)	3	3 (PP=0.55)	3 (PP=0.83)	3 (PP=1.00)
	KU983760							
	KU983761		4 (PP=0.91)	4 (PP=0.87)	4	4 (PP=0.55)	4 (PP=0.83)	4 (PP=1.00)
	KU983762							
Nanshanentulus urumchiensis	HQ882824	1	1 (PP=1.00)	1 (PP=1.00)	1	-	-	-
Verrucoentomon louisanne	KX620026	1	1 (PP=1.00)	1 (PP=1.00)	1	-	-	-
Verrucoentomon anatoli	KX620021-25	1	1 (PP=1.00)	1 (PP=1.00)	1	-	-	-
Verrucoentomon yushuensis	MK636821	1	1 (PP=1.00)	1 (PP=1.00)	1	-	-	-
Huashanentulus huashanensis	HQ882821	1	1 (PP=1.00)	1 (PP=1.00)	1	-	-	-
Nosekiella sinensis	HQ882823	1	1 (PP=1.00)	1 (PP=1.00)	1	-	-	-
Acerentomon dispar	PROTA001-15; PROTA002-15	1	1 (PP=1.00)	1 (PP=1.00)	1	1 (PP=0.98)	1 (PP=0.98)	1 (PP=1.00)
	PROTA023-15							
	PROTA013-15							
	PROTA008-15; PROTA009-15	2	2 (PP=1.00)	2 (PP=1.00)	2	2 (PP=0.69)	2 (PP=0.90)	2 (PP=0.99)
	PROTA021-15; PROTA025-15	3	3 (PP=1.00)	3 (PP=1.00)	3	3 (PP=0.69)	3 (PP=0.90)	3 (PP=0.99)
Acerentomon microrhinus	PROAT022-12	1	1 (PP=1.00)	1 (PP=1.00)	1	1 (PP=0.81)	1 (PP=0.94)	1 (PP=0.99)
	JQ728012	2	2 (PP=1.00)	2 (PP=1.00)	2	2 (PP=0.76)	2 (PP=0.94)	2 (PP=0.99)
	HQ882819	3	3 (PP=1.00)	3 (PP=1.00)	3	3 (PP=0.81)	3 (PP=0.95)	3 (PP=0.99)
Acerentulus confinis	ABPE004-17	1	1 (PP=1.00)	1 (PP=1.00)	1	-	-	-
Acerentulus charrieri	GBA8988-12; GBA8987-12	1	1 (PP=1.00)	1 (PP=1.00)	1	-	-	-
Acerentulus sinensis	HQ882820	1	1 (PP=1.00)	1 (PP=1.00)	1	-	-	-
Sinentomon erythranum	HQ882826	1	1 (PP=1.00)	1 (PP=1.00)	1	-	-	-
Kenyentulus japonicus	MK636822	1	1 (PP=1.00)	1 (PP=1.00)	1	-	-	-
Anisentomon magnispinosum	MK636823	1	1 (PP=1.00)	1 (PP=1.00)	1	-	-	-
Eosentomidae sp.	MG319097	1	1 (PP=1.00)	1 (PP=1.00)	1	1 (PP=0.97)	1 (PP=0.95)	1 (PP=1.00)
	MG318856; MG316089	2	2 (PP=1.00)	2 (PP=0.98)	2	2 (PP=0.98)	2 (PP=0.98)	2 (PP=1.00)
	MG319651							
	MG320620							
	MG318042	3	3 (PP=1.00)	3 (PP=1.00)	3	3 (PP=.98)	3 (PP=0.95)	3 (PP=1.00)
Anisentomon chinensis	MK636824	1	1 (PP=1.00)	1 (PP=1.00)	1	-	-	-
Zhongguhentomon piligeroum	HQ882828	1	1 (PP=1.00)	1 (PP=1.00)	1	-	-	-
Eosentomon communis	MK636825	1	1 (PP=1.00)	1 (PP=1.00)	1	-	-	-
Eosentomon nivicolum	MK636825	1	1 (PP=1.00)	1 (PP=1.00)	1	-	-	-
Andinentulus rapoporti	KJ395311; KJ395313	1	1 (PP=1.00)	1 (PP=0.98)	1	1 (PP=1.00)	1 (PP=0.97)	1 (PP=1.00)
	KJ395312							
Huhentomon plicatunguis	HQ882813	1	1 (PP=1.00)	1 (PP=1.00)	1	-	-	-
Ionescuellum haybache	PROAT043-12	1	1 (PP=1.00)	1 (PP=1.00)	1	1 (PP=0.90)	1 (PP=0.94)	1 (PP=1.00)
	PROAT008-12	2	2 (PP=1.00)	2 (PP=1.00)	2	2 (PP=0.89)	2 (PP=0.96)	2 (PP=1.00)
	PROAT002-12; PROAT009-12 to 011-12; PROAT013-12; PROAT014-12							
Proturentomon chinense	HQ882814	1	1 (PP=1.00)	1 (PP=1.00)	1	-	-	-
Ionescuellum carpaticum	PROAT004-12	1+2	1 (PP=1.00)	1 (PP=1.00)	1	* 1+2 (PP=0.50); * 1 (PP=0.41)	1 (PP=0.71)	1 (PP=1.00)
	PROAT007-12		2 (PP=1.00)	2 (PP=1.00)	2	*2 (PP=0.41)	2 (PP=0.71)	2 (PP=1.00)
Condeellum ishiianum	MK636827	1	1 (PP=1.00)	1 (PP=1.00)	1	-	-	-
Neocondeellum dolichotarsum	HQ882815	1	1 (PP=1.00)	1 (PP=1.00)	1	-	-	-
Hesperentomon bolense	MF918363; MF918365-70	1+2	1 (PP=1.00)	1 (PP=1.00)	1	1+2 (PP=0.56)	1 (PP=0.67)	1 (PP=1.00)
	MF918364		2 (PP=1.00)	2 (PP=1.00)	2		2 (PP=0.67)	2 (PP=1.00)
Hesperentomon yangi	KF530825	1	1 (PP=0.93)	1 (PP=1.00)	1	1 (PP=0.98)	1 (PP=0.98)	1 (PP=1.00)
	KF530824							
	KF530826							
Hesperentomon perctigastrulum	HQ882812	1	1 (PP=1.00)	1 (PP=1.00)	1	1 (PP=0.86)	1 (PP=0.94)	1 (PP=1.00)
	MF918361-62	2	2 (PP=1.00)	2 (PP=1.00)	2	2 (PP=0.86)	2 (PP=0.95)	2 (PP=1.00)
Hesperentomon chianghaiensis	MF918360	1	1 (PP=1.00)	1 (PP=1.00)	1	-	-	-
Hesperentomon xiningense	KF530827	1	1 (PP=1.00)	1 (PP=1.00)	1	-	-	-

•

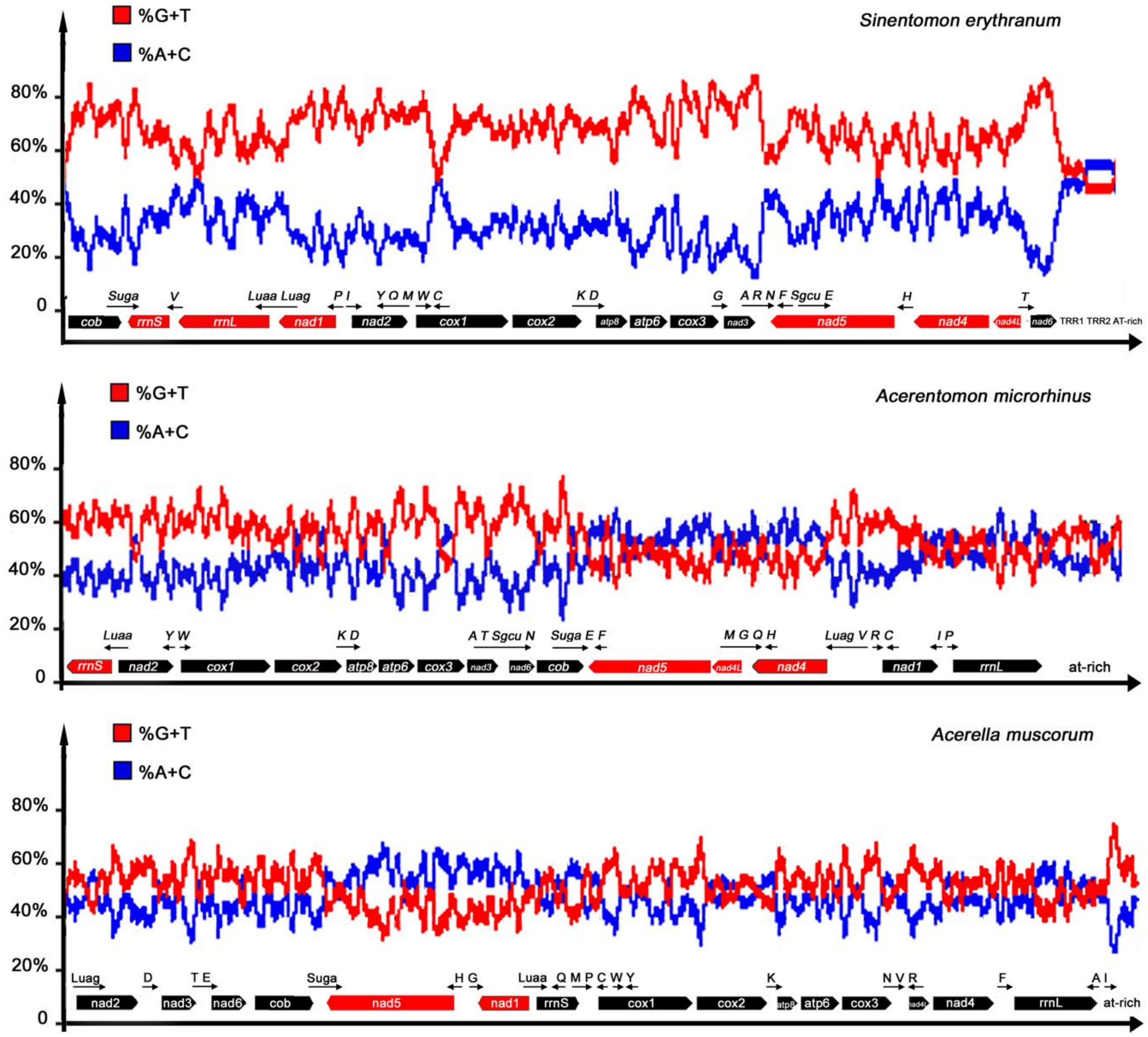

Figure S1. G+T *vs*. A+C content, calculated in a sliding window of 100 bp along the J-strand of the genomes of *S. erythranum*, *A. muscorum* and *A. microrhinus*. Genome maps are shown below each graph.

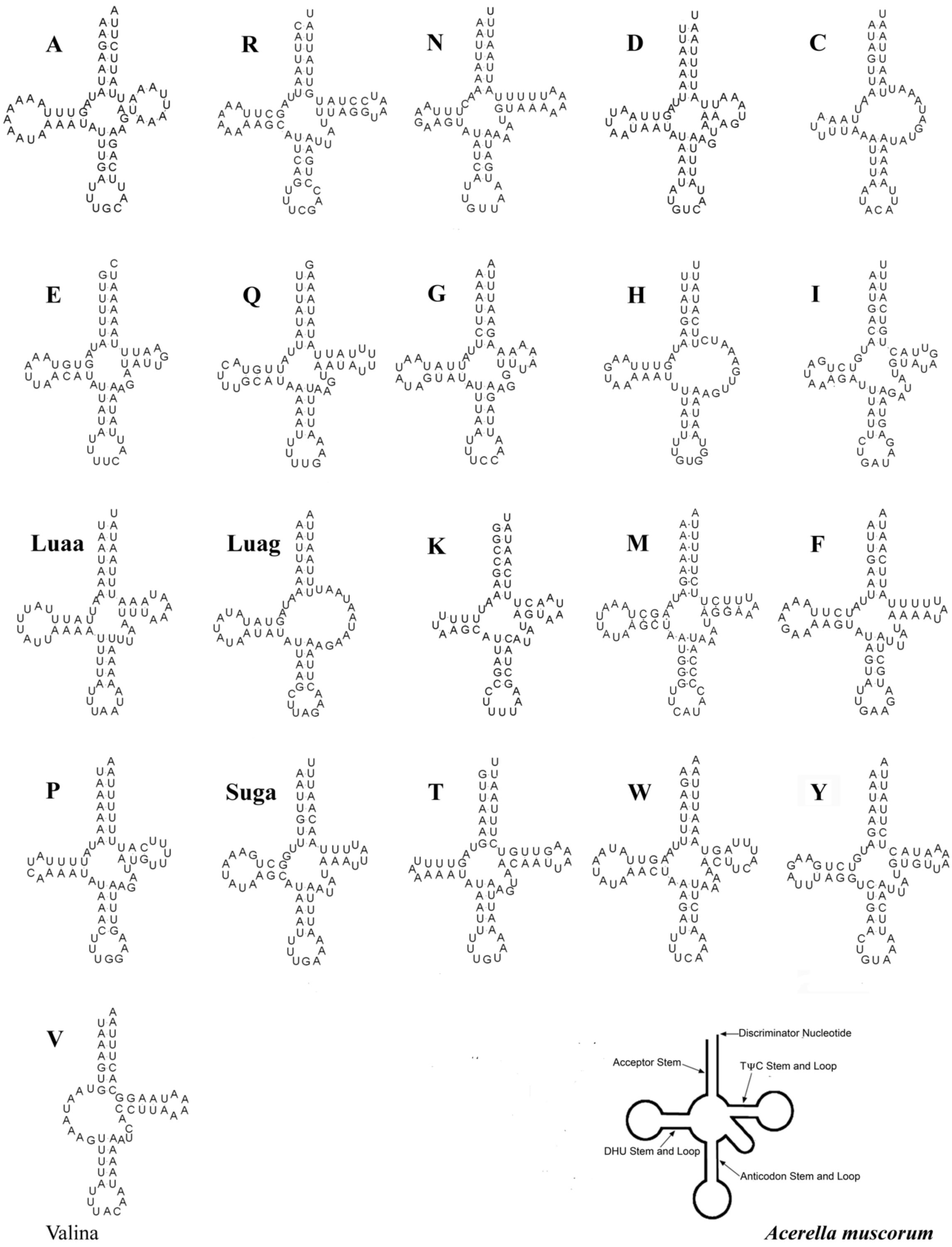

Figure S2. Secondary structure of the 21 tRNAs detected in the mitochondrial genome of *A. muscorum*. The *trnSgcu* was not identified.

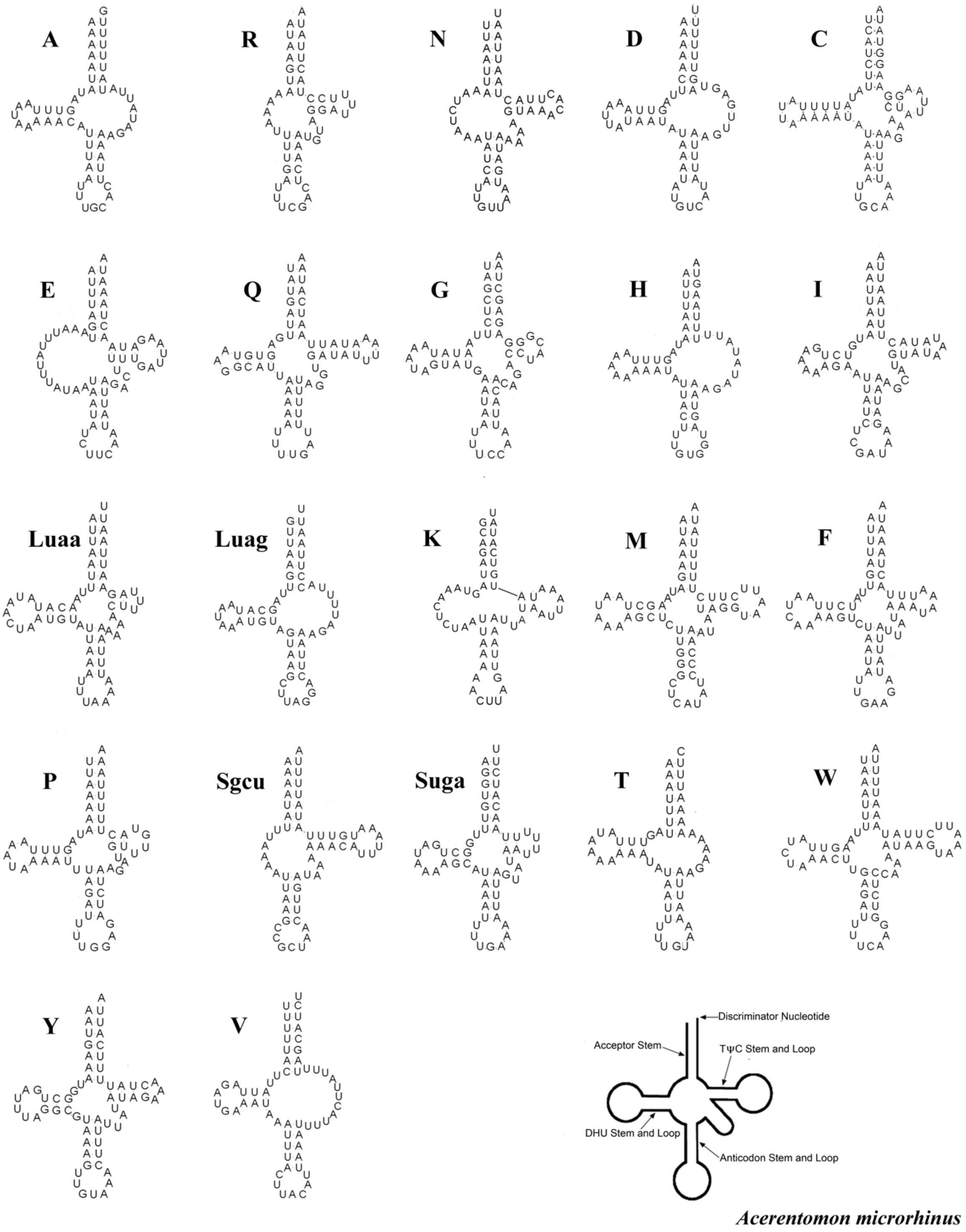

Figure S3. Secondary structure of the 22 tRNAs detected in the mitochondrial genome of *A. microrhinus*.

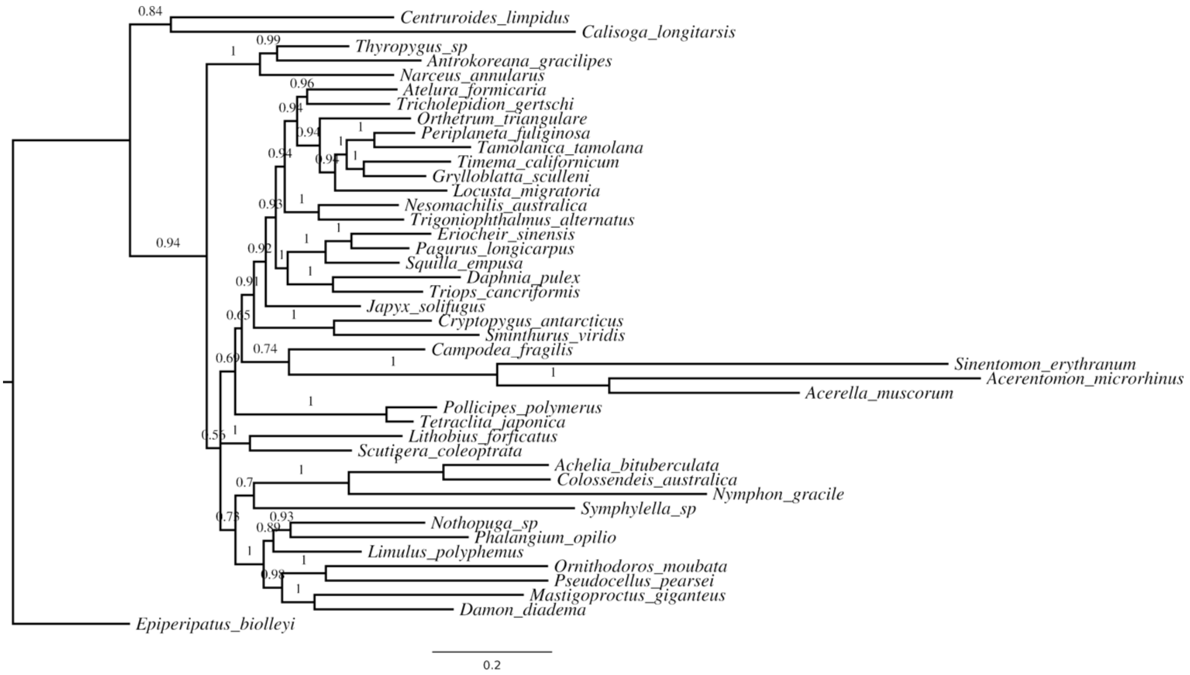
Centruroides_limpidus
Calisoga_longitarsis
Thyropygus_sp
Antrokoreana_gracilipes
Narceus_annularus
Atelura_formicaria
Tricholepidion_gertschi
Orthetrum_triangulare
Periplaneta_fuliginosa
Tamolanica_tamolana
Timema_californicum
Grylloblatta_sculleni
Locusta_migratoria
Nesomachilis_australica
Trigoniophthalmus_alternatus
Eriocheir_sinensis
Pagurus_longicarpus
Squilla_empusa
Daphnia_pulex
Triops_cancriformis
Japyx_solifugus
Cryptopygus_antarcticus
Sminthurus_viridis
Campodea_fragilis
Sinentomon_erythranum
Acerentomon_microrhinus
Acerella_muscorum
Pollicipes_polymerus
Tetraclita_japonica
Lithobius_forficatus
Scutigera_coleoptrata
Achelia_bituberculata
Colossendeis_australica
Nymphon_gracile
Symphylella_sp
Nothopuga_sp
Phalangium_opilio
Limulus_polyphemus
Ornithodoros_moubata
Pseudocellus_pearsei
Mastigoproctus_giganteus
Damon_diadema
Epiperipatus_biolleyi
0.2

(a)

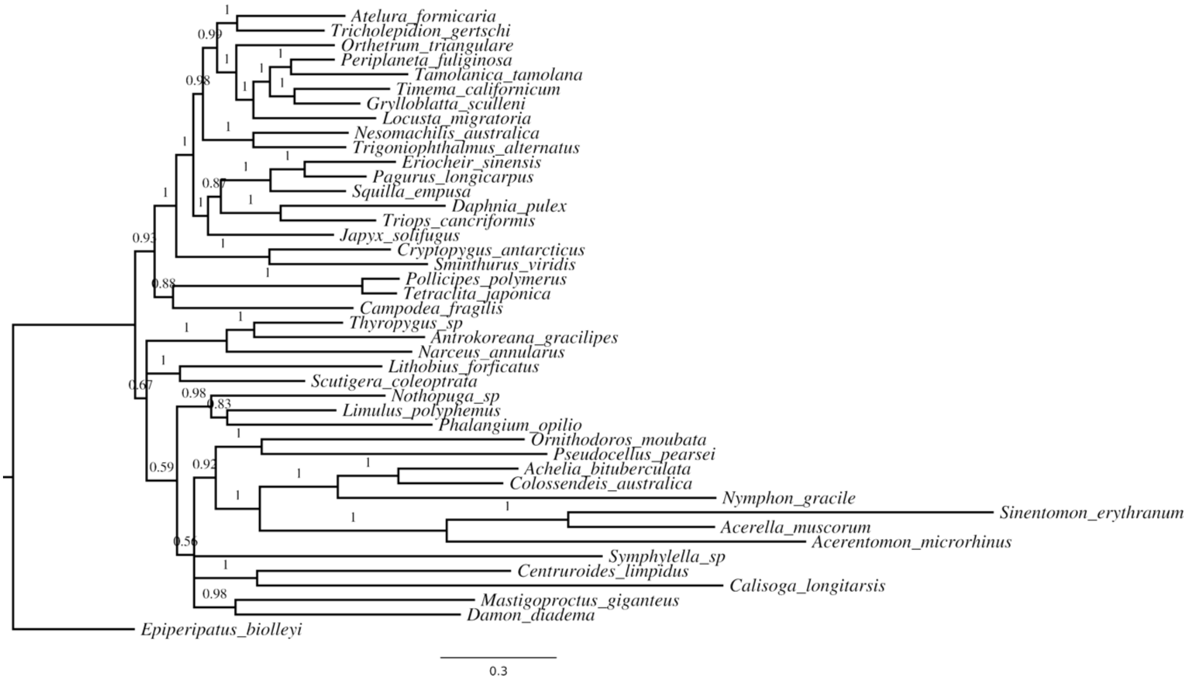
Atelura_formicaria
Tricholepidion_gertschi
Orthetrum_triangulare
Periplaneta_fuliginosa
Tamolanica_tamolana
Timema_californicum
Grylloblatta_sculleni
Locusta_migratoria
Nesomachilis_australica
Trigoniophthalmus_alternatus
Eriocheir_sinensis
Pagurus_longicarpus
Squilla_empusa
Daphnia_pulex
Triops_cancriformis
Japyx_solifugus
Cryptopygus_antarcticus
Sminthurus_viridis
Pollicipes_polymerus
Tetraclita_japonica
Campodea_fragilis
Thyropygus_sp
Antrokoreana_gracilipes
Narceus_annularus
Lithobius_forficatus
Scutigera_coleoptrata
Nothopuga_sp
Limulus_polyphemus
Phalangium_opilio
Ornithodoros_moubata
Pseudocellus_pearsei
Achelia_bituberculata
Colossendeis_australica
Nymphon_gracile
Sinentomon_erythranum
Acerella_muscorum
Acerentomon_microrhinus
Symphylella_sp
Centruroides_limpidus
Calisoga_longitarsis
Mastigoproctus_giganteus
Damon_diadema
Epiperipatus_biolleyi
0.3

(b)

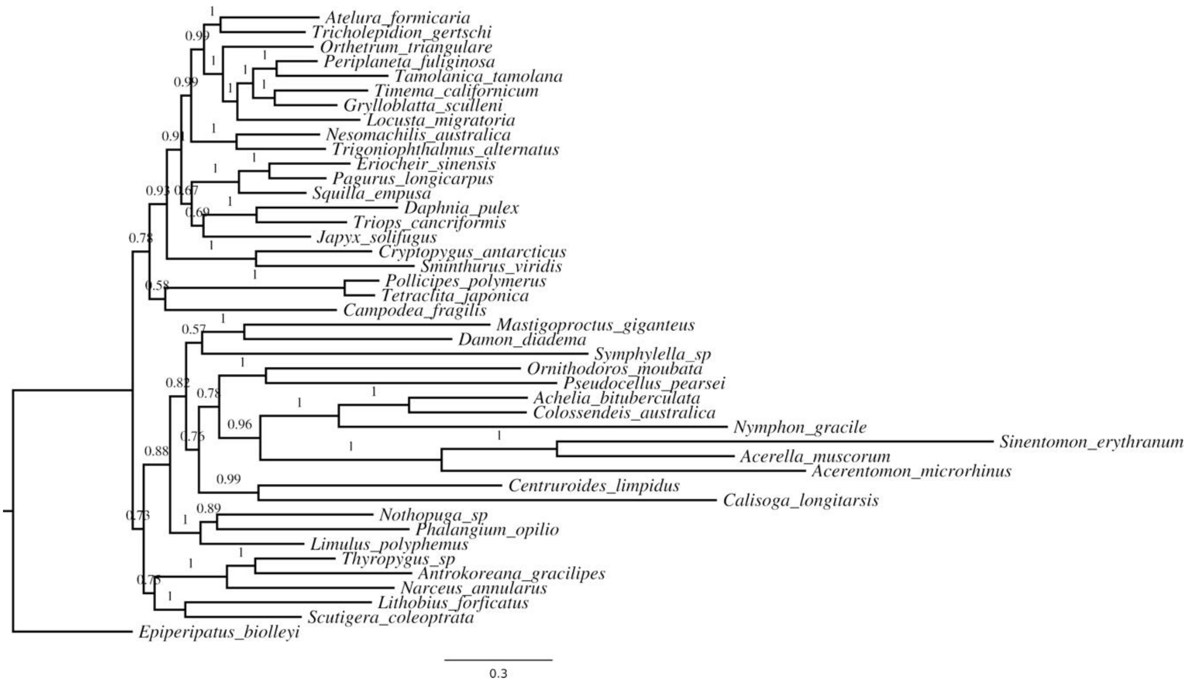

(c)

Figure S4. Phylogenetic analysis of Protura in the context of Arthropoda based on a subset of mitochondrial genes found in the same orientation. Posterior probabilities are shown above nodes. a: nucleotide dataset; b: amino acid dataset, model mixed; c: amino acid dataset, model MtPAN.

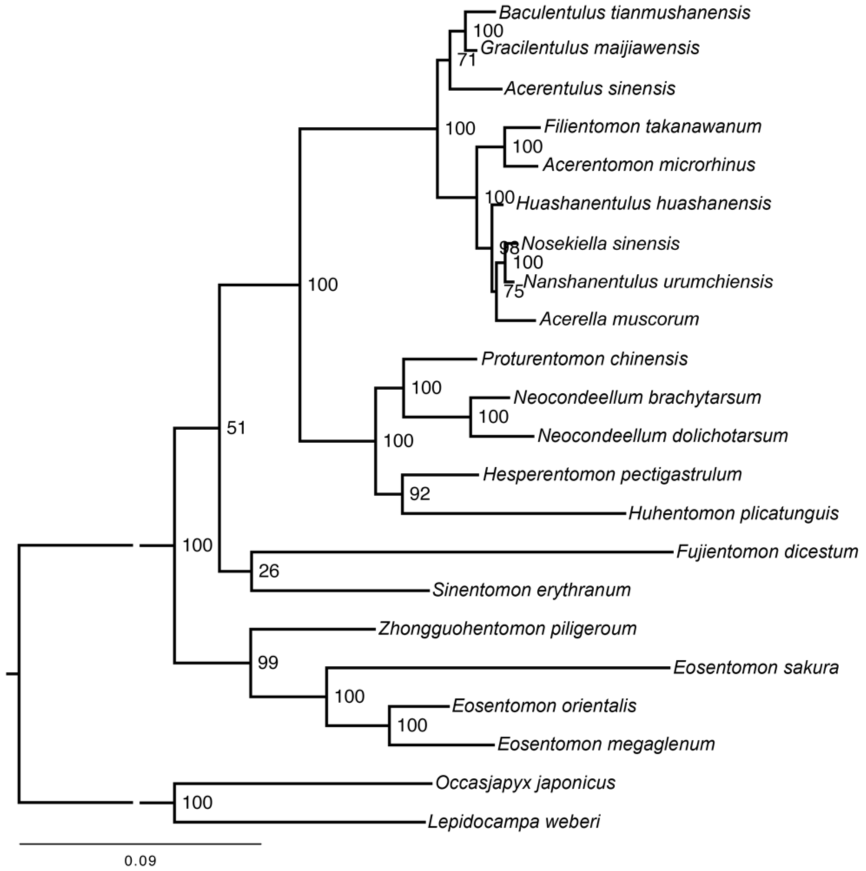

Figure S5. Phylogenetic analysis of Protura based on the reduced *18S* and *28S* data set. Boostrap values shown at nodes.
